# Development and Validation of a Pathomics Model for Prognosis Prediction in Neoadjuvant Therapy‐Treated Breast Cancer: A Retrospective, Multicenter Study

**DOI:** 10.1002/mco2.70826

**Published:** 2026-07-02

**Authors:** Yani Wei, Wei Lou, Zongbo Han, Fan Yang, Fengling Li, Haofeng Li, Huijuan Shi, Bing Wei, Hongjun Li, Yuanyuan Zhao, Xiuli Xiao, Yongquan Yang, Anjia Han, Jianhua Yao, Hong Bu

**Affiliations:** ^1^ Department of Pathology, West China Hospital Sichuan University Chengdu Sichuan China; ^2^ Institute of Clinical Pathology, West China Hospital Sichuan University Chengdu Sichuan China; ^3^ Department of Pathology, The First Affiliated Hospital Sun Yat‐sen University Guangzhou Guangdong China; ^4^ College of Mathematical Medicine Zhejiang Normal University Jinhua Zhejiang China; ^5^ State Key Laboratory of Networking and Switching Technology Beijing University of Posts and Telecommunications Beijing China; ^6^ Tencent AI Lab Shenzhen Guangdong China; ^7^ School of Systems Science and Engineering Sun Yat‐sen University Guangzhou Guangdong China; ^8^ Department of Pathology, Shanxi Province Cancer Hospital/Shanxi Hospital Affiliated to Cancer Hospital Chinese Academy of Medical Sciences/Cancer Hospital Affiliated to Shanxi Medical University Taiyuan Shanxi China; ^9^ Department of Pathology The Affiliated Hospital of Southwest Medical University Luzhou Sichuan China

**Keywords:** breast cancer, deep learning, neoadjuvant therapy, prognosis, whole‐slide image

## Abstract

Accurate outcome prediction is essential for personalized therapy in patients with breast cancer receiving neoadjuvant therapy (NAT). In this study, we developed and validated a multimodal artificial intelligence model, the ClinicHistomics Integrated Outcome Prediction Model (CIOPM), to predict prognosis in NAT‐treated breast cancer patients by integrating clinicopathological data and hematoxylin and eosin (H&E)‐stained surgical specimen whole‐slide images (WSIs). A total of 847 WSIs from 835 patients across four multicenter cohorts collected between January 2008 and May 2020 were enrolled in model development and external validation. The CIOPM demonstrated robust predictive performance for overall survival (OS) and disease‐free survival (DFS) in the two validation cohorts (VCs), yielding C‐indices of 0.933 (95% CI: 0.878–0.977) and 0.915 (95% CI: 0.850–0.960) for OS in VC 1, and 0.947 (95% CI: 0.895–0.983) and 0.937 (95% CI: 0.905–0.965) for DFS in VC 2, respectively. The CIOPM enables accurate stratification of patients with NAT‐treated breast cancer into high‐ and low‐risk groups, achieving an AUC of 0.957 for both OS and DFS prediction. Moreover, it demonstrated superior performance in ablation studies, subgroup analyses, and clinical risk model comparisons. This model provides reliable risk stratification and personalized treatment decisions.

## Graphical abstract

We retrospectively enrolled breast cancer patients receiving neoadjuvant therapy from multiple centers. We analyzed digital whole‐slide images, extracted pathological features, and built integrated prediction models combining pathological features and text information. The established model was fully validated via multiple statistical methods in external cohorts for reliable clinical outcome prediction.

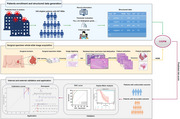

## 1 Introduction

The standard treatment for patients with breast cancer includes neoadjuvant therapy (NAT), which induces substantial tumor downsizing and downstaging, followed by either modified radical mastectomy or breast‐conserving surgery and adjuvant chemotherapy or radiotherapy [1]. Previous studies have demonstrated that patients with triple‐negative and HER2+ breast cancer, in particular, who achieve pCR after NAT, have significantly improved overall survival (OS) and disease‐free survival (DFS) [2, 3]. These patients might benefit from organ‐preserving and function‐preserving strategies, such as local excision, watching, and waiting [1]. Although pCR has become a surrogate prognostic factor for survival following therapy, some patients still experience distant metastases or even death after obtaining pCR [4, 5, 6]. Identifying high‐risk patients who may have an unfavorable OS or DFS and providing them with personalized treatment strategies could improve their quality of life and long‐term survival. In contrast, postoperative adjuvant chemoradiotherapy may be an alternative course of treatment for patients who may have a favorable outcome, and the toxicity of increased therapy should be considered. Therefore, a reliable prognostic tool is urgently needed to guide adjuvant therapy decisions for individualized postoperative treatment of patients with breast cancer treated with NAT.

Previous research has focused on predicting the response of NAT for breast cancer patients to determine the relationship between a patient's sensitivity to chemotherapeutic agents and prognosis; however, indirect outcome prediction may lead to inaccurate risk assessment [7]. Although preoperative core needle biopsy tissue images are the primary source of information for studies investigating pathological image biomarkers for pCR forecasting [8], these images may not be as representative for predicting chemotherapeutic agent response for heterogeneous tumors. In addition, the various effects of NAT drugs on the tumor can result in complex and varied changes in the histological morphology of the tumor, which may directly correlate with patient prognosis. However, accurately predicting prognosis based solely on morphological changes observed under a microscope is difficult for pathologists. Postoperative pathological images may provide useful information about patient prognosis, and the use of representative postoperative images may greatly enhance prognostic prediction.

Significant advancements in artificial intelligence (AI) for medical applications have been made in the last few years, especially in the areas of precision diagnosis, computer‐aided screening and triage, and decision support [9, 10]. Precise and reliable prediction models from histology images could be created with the help of the burgeoning field of digital pathology [11]. In addition, the application of machine learning (ML) techniques for the qualitative and quantitative analysis of histological images may address tumor heterogeneity and observer variation. Deep neural networks (DNNs) are common deep learning (DL) architectures that help identify features in medical images [12]. Preliminary data indicate that ML algorithms can accurately detect local or global variations that might go undetected by a pathologist [13].

Integrating multimodal data could improve the predictive ability of current models and supplement tumor heterogeneity at various scales [14]. Oncologic prognostication commonly involves the integration of histopathological, clinical, and genomic markers, which contribute to variability in therapeutic response and clinical outcomes [15]. To determine an accurate diagnosis, prognosis, and course of treatment for each patient, doctors in clinical practice typically consult extensive information derived from multimodality data [16]. Integrating data from various modalities may increase the prediction model's robustness and accuracy, suggesting its potential for clinical application. In a preliminary study, we confirmed that the histomics of tumor and stroma could enhance the prediction of NAT response in patients with breast cancer [8, 17]. However, these results require further optimization and prospective validation in multicenter datasets to verify the reproducibility of this strategy. To confirm the reproducibility and robustness of this approach in clinical practice, additional optimization and integration in multicenter datasets are necessary to validate these results.

In this study, we aimed to develop and validate the ClinicHistomics Integrated Outcome Prediction Model (CIOPM) for forecasting OS and DFS in NAT‐treated patients with breast cancer by combining whole‐slide images (WSIs) of surgical specimens stained with hematoxylin and eosin (H&E) and tabular clinical data.

## 2 Results

### 2.1 Participant Characteristics

For CIOPM development and validation, data were retrospectively collected from four hospitals: West China Hospital of Sichuan University (WCH‐SU, Chengdu, China), the First Affiliated Hospital of Sun Yat‐sen University (FAH‐SYSU, Guangzhou, China), the Chinese Academy of Medical Sciences/Cancer Hospital Affiliated to Shanxi Medical University (CHA‐SMU, Taiyuan, China), and the Affiliated Hospital of Southwest Medical University (AFH‐SMU, Luzhou, China).

**FIGURE 1:**
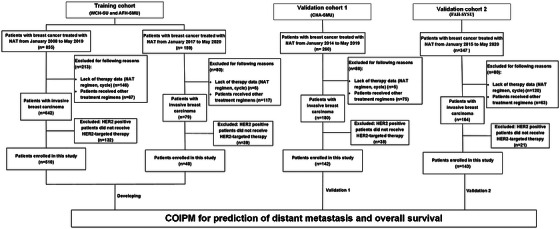
Flowchart of patient selection and study design. This diagram illustrates the selection process for the training cohort (WCH‐SU and AFH‐SMU) and two external validation cohorts (CHA‐SMU and FAH‐SYSU). The exclusion criteria included the lack of neoadjuvant therapy data and receipt of non‐anthracycline and non‐paclitaxel treatment regimens. The final cohort comprised 550 patients (561 slides) in the training set, 142 patients (142 slides) in VC 1, and 143 patients (144 slides) in VC 2. The clinicopathological‐image‐integrated outcome prediction model was developed using the training cohort and externally validated for predicting DFS and OS. HER2, human epidermal growth factor receptor 2; *n*, number; NAT, neoadjuvant therapy.

This study included 835 patients with 847 slides who received NATs from four centers, including 510 patients with 521 slides from the WCH‐SU, 143 patients with 144 slides from FAH‐SYSU, 142 patients with 142 slides from the CHA‐SMU, and 40 patients with 40 slides from the AFH‐SMU (Figure 1). The TC included 550 patients with 561 slides and were from WCH‐SU and AFH‐SMU from January 1, 2008 to May 31, 2019; the validation Cohort 1 (VC 1) included 142 patients with 142 slides and was from CHA‐SMU from January 1, 2014 to May 31, 2019; and the validation Cohort 2 (VC 2) included 143 patients with 144 slides and was from FAH‐SYSU from January 1, 2015 to May 31, 2020.

Table 1 presents the characteristics of the enrolled patients. The mean follow‐up durations for OS were 52.3, 33.9, and 63.4 months in TC, VC 1, and VC 2; the mean follow‐up times for DFS were 41.8, 32.6, and 58.9 months in TC, VC 1, and VC 2.

**TABLE 1:** Baseline characteristics in the training and validation cohorts.

Characteristics	WCH‐SU and AFH‐SMU, TC (*n* = 601)	CHA‐SMU, VC 1 (*n* = 142)	FAH‐SYSU, VC 2 (*n* = 143)
Age (years, mean ± SD)	47.5 ± 9.8	49.3 ± 10.0	46.8 ± 9.5
Menopausal status			
Premenopausal	339 (61.6%)	101 (71.1%)	104 (72.7%)
Postmenopausal	211 (38.4%)	41 (28.9%)	39 (27.3%)
Laterality			
Left	274 (49.8%)	89 (62.7%)	81 (56.6%)
Right	276 (50.2%)	53 (37.3%)	62 (43.4%)
NAT type			
Anthracycline	33 (6.0%)	16 (11.3%)	4 (2.8%)
Paclitaxel	84 (15.3%)	6 (4.2%)	10 (7.0%)
Anthracycline and paclitaxel	433 (78.7%)	120 (84.5%)	129 (90.2%)
pCR or non‐pCR			
pCR	131 (23.8%)	35 (24.6%)	23 (16.1%)
non‐pCR	419 (76.2%)	107 (75.4%)	120 (83.9%)
cTNM stage			
I+II	163 (29.6%)	0 (0.0%)	73 (51.0%)
III	374 (68.0%)	72 (50.7%)	70 (49.0%)
Unknown	13 (2.4%)	70 (49.3%)	0 (0.0%)
pT			
T0	39 (7.1%)	4 (2.8%)	24 (16.8%)
T1/T2	452 (82.2%)	130 (91.5%)	80 (55.9%)
T3/T4	59 (10.7%)	8 (5.6%)	28 (19.6%)
pN			
N0	168 (30.5%)	50 (35.2%)	59 (41.3%)
N1/N2	301 (54.7%)	68 (47.9%)	72 (50.3%)
N3	81 (14.7%)	24 (16.9%)	12 (8.4%)
ER (pre)			
Positive	402 (73.1%)	106 (74.6%)	53 (37.1%)
Negative	147 (26.7%)	36 (25.4%)	90 (62.9%)
Unknown	1 (0.2%)	0 (0.0%)	0 (0.0%)
PR (pre)			
Positive	374 (68.0%)	93 (65.5%)	63 (44.1%)
Negative	175 (31.8%)	49 (34.5%)	80 (55.9%)
Unknown	1 (0.2%)	0 (0.0%)	0 (0.0%)
HER2 (pre)			
Positive	131 (23.8%)	20 (14.1%)	56 (39.2%)
Negative	419 (76.2%)	122 (85.9%)	87 (60.8%)
Ki67 (pre)			
≤ 20%	107 (19.5%)	22 (15.5%)	30 (21.0%)
> 20%	418 (76.0%)	119 (83.8%)	113 (79.0%)
Unknown	25 (4.5%)	1 (0.7%)	0 (0.0%)
Subtype (pre)			
HR+/HER2−	335 (60.9%)	103 (72.5%)	65 (45.5%)
HR+/HER2+	103 (18.7%)	4 (2.8%)	31 (21.7%)
HR−/HER2+	28 (5.1%)	16 (11.3%)	25 (17.5%)
HR−/HER2−	83 (15.1%)	19 (13.4%)	22 (15.4%)
Unknown	1 (0.2%)	0 (0.0%)	0 (0.0%)
ER (post)			
Positive	339 (61.6%)	3 (2.1%)	71 (49.7%)
Negative	111 (20.2%)	2 (1.4%)	42 (29.4%)
Unknown	100 (18.2%)	0 (0.0%)	30 (20.0%)
PR (post)			
Positive	312 (56.7%)	3 (2.1%)	56 (39.2%)
Negative	137 (24.9%)	2 (1.4%)	56 (39.2%)
Unknown	101 (18.4%)	0 (0.0%)	31 (21.6%)
HER2 (post)			
Positive	87 (15.8%)	1 (0.7%)	34 (23.8%)
Negative	307 (55.8%)	2 (1.4%)	76 (53.1%)
Unknown	156 (28.4%)	139 (97.9%)	33 (23.1%)
Ki67 (post)			
< 20%	196 (35.6%)	3 (2.1%)	64 (44.8%)
≥ 20%	219 (39.8%)	2 (1.4%)	49 (34.3%)
Unknown	135 (24.5%)	137 (96.5%)	30 (20.9%)
LVI			
Present	122 (22.2%)	55 (38.7%)	22 (15.4%)
Absent	424 (77.1%)	87 (61.3%)	121 (84.6%)
Unknown	4 (0.7%)	0 (0.0%)	
Subtype (post) Molecular Classification Molecular Classification			
HR+/HER2−	275 (50.0%)	2 (1.4%)	53 (37.1%)
HR+/HER2+	61 (11.1%)	1 (0.7%)	18 (12.6%)
HR−/HER2+	26 (4.7%)	0 (0.0%)	16 (11.2%)
HR−/HER2−	69 (12.5%)	0 (0.0%)	23 (16.1%)
Unknown	119 (21.6%)	0 (0.0%)	33 (23.0%)
sTILs			
< 20%	492 (89.5%)	122 (85.9%)	143 (100.0%)
≥ 20%	47 (8.5%)	20 (14.1%)	0 (0.0%)
Unknown	11 (2.0%)	0 (0.0%)	0 (0.0%)
iTILs			
< 10%	511 (92.9%)	108 (76.1%)	88 (61.5%)
≥ 10%	28 (5.1%)	34 (23.9%)	55 (38.5%)
Unknown	11 (2.0%)	0 (0.0%)	0 (0.0%)
Radiotherapy			
Yes	264 (48.0%)	97 (68.3%)	102 (71.3%)
No	286 (52.0%)	44 (31.0%)	41 (28.7%)
Endocrine therapy			
Yes	342 (62.2%)	86 (50.6%)	80 (55.9%)
No	208 (37.8%)	56 (39.4%)	63 (44.1%)
Anti‐HER2 therapy			
Yes	145 (26.4%)	12 (8.5%)	57 (39.9%)
No	405 (73.6%)	130 (91.5%)	86 (60.1%)
Surgery type			
Mastectomy	528 (96.0%)	138 (97.2%)	143 (100.0%)
Lumpectomy	13 (4.0%)	4 (2.8%)	0 (0.0%)
Unknown	9 (%)	0 (0.0%)	0 (0.0%)
Follow‐up time			
OS (months, mean ± SD)	52.3 ± 30.2	33.9 ± 16.3	63.4 ± 18.2
DFS (months, mean ± SD)	41.8 ± 29.1	32.6 ± 16.4	58.9 ± 21.2
RCB			
0	36 (6.5%)	3 (2.1%)	—
I	22 (4.0%)	7 (4.9%)	—
II	222 (40.4%)	73 (51.4%)	—
III	203 (36.9%)	59 (41.5%)	—
Unknown	67 (12.2%)	0 (0.0%)	—
AJCC			
0	35 (6.4%)	0 (0.0%)	23 (16.1%)
I	71 (12.9%)	3 (2.1%)	25 (17.5%)
II	228 (41.5%)	69 (48.6%)	72 (50.3%)
III	216 (39.3%)	70 (49.3%)	23 (16.1%)
Neo‐Bioscore			
0	1 (0.2%)	0 (0.0%)	—
1	13 (2.4%)	5 (3.5%)	—
2	47 (8.5%)	22 (15.5%)	—
3	121 (22.0%)	63 (44.4%)	—
4	149 (27.1%)	39 (27.5%)	—
5	43 (7.8%)	11 (7.7%)	—
6	8 (1.5%)	2 (1.4%)	—
Unknown	168 (30.5%)	0 (0.0%)	—

Abbreviations: AJCC, the American Joint Committee on Cancer; ER, estrogen receptor; HER2, human epidermal growth factor receptor 2; iTILs, intra‐tumoral tumor‐infiltrating lymphocytes; LVI, lymphovascular invasion; NAT, neoadjuvant therapy; pCR, pathological complete response; pN, pathologic node stage; post, post‐NAT; PR, progesterone receptor; pre, pre‐NAT; pT, pathologic tumor stage; RCB, residual cancer burden; sTILs, stromal tumor‐infiltrating lymphocytes; TNM, tumor‐node‐metastasis stage.

### 2.2 Development and Validation of CIOPM

A total of 847 slides from four institutions were used for model development and validation: TC from WCH‐SU and AFH‐SMU, VC 1 from CHA‐SMU, and VC 2 from FAH‐SYSU. The distribution of the number of 1024 × 1024 patches at 40× magnification is presented in Figure S1.

**FIGURE 2:**
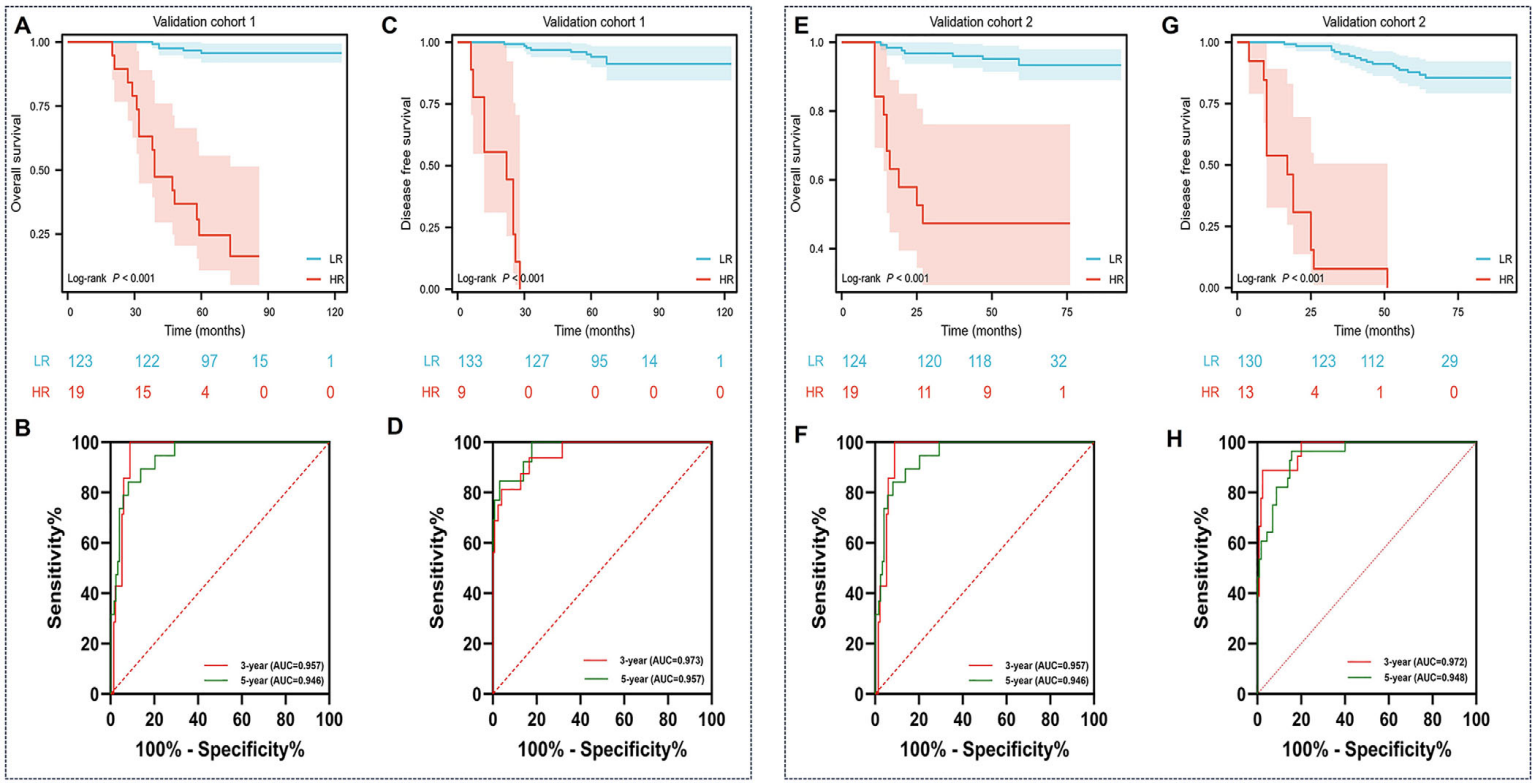
Prognostic stratification and predictive performance of CIOPM in validation cohorts. (A and E) Kaplan–Meier curves for OS stratified by CIOPM risk groups in VC 1 and VC 2, respectively. (B and F) Time‐dependent ROC curves for 3‐ and 5‐year OS prediction by CIOPM in VC 1 and VC 2, respectively. (C and G) Kaplan–Meier curves for DFS stratified by CIOPM risk groups in VC 1 and VC 2, respectively. (D and H) Time‐dependent ROC curves for 3‐ and 5‐year DFS prediction by CIOPM in VC 1 and VC 2, respectively. AUC, area under the curve; CIOPM, ClinicHistomics Integrated Outcome Prediction Model; DFS, disease‐free survival; HR, high‐risk; LR, low‐risk; OS, overall survival; ROC, receiver operating characteristic; VC 1, validation Cohort 1; VC 2, validation Cohort 2.

The CIOPM demonstrated excellent prognostic performance in two independent validation cohorts (VC 1 and VC 2). For OS, the concordance index (C‐index) of CIOPM was 0.933 (95% CI: 0.878–0.977) in VC 1 and 0.915 (95% CI: 0.850‐0.960) in VC 2. For DFS, the C‐indexes were 0.947 (95% CI: 0.895–0.983) in VC 1 and 0.937 (95% CI: 0.905–0.965) in VC 2 (Table S1). As illustrated in Figure 2, the CIOPM effectively stratified patients into high‐risk and low‐risk groups in both cohorts. In VC 1, Kaplan–Meier analysis revealed significantly superior OS (Figure 2A) and DFS (Figure 2C) in the LR group compared with the HR group (both log‐rank *p* < 0.001). Similarly, in VC 2, the LR group exhibited markedly better OS (Figure 2E) and DFS (Figure 2G) than the HR group (both log‐rank *p* < 0.001). Table S2A,B shows the corresponding risk values for each patient predicted by CIOPM in VC 1. Table S3A,B shows the corresponding risk values for each patient predicted by CIOPM in VC 2. Time‐dependent receiver operating characteristic (ROC) analysis further confirmed the predictive accuracy of the model. In VC 1, the area under the curve (AUC) values for 3‐ and 5‐year OS were 0.957 (Figure 2B) and 0.946 (Figure 2D), respectively. Consistent results were observed in VC 2, with AUC values of 0.957 (Figure 2F) for 3‐year OS and 0.948 (Figure 2H) for 5‐year DFS, indicating robust and consistent predictive performance across both validation cohorts.

### 2.3 Results of the Ablation Study

We conducted ablation experiments on two independent external validation cohorts (CHA‐SMU and FAH‐SYSU) by evaluating model performance using each modality alone to clarify the individual contributions of pathological images and clinical information. On VC 1 (CHA‐SMU), the clinical information‐only models (CIMs) achieved C‐indexes of 0.873 for OS and 0.901 for DFS, while the pathological image‐only models (PIMs) yielded 0.687 (OS) and 0.597 (DFS) (Table S1). On VC 2 (FAH‐SYSU), CIMs reached C‐indexes of 0.853 (OS) and 0.849 (DFS), compared with 0.637 (OS) and 0.646 (DFS) for PIMs. In contrast, the multimodal integration substantially improved performance, with the combined model achieving C‐indexes of 0.926 (OS) and 0.955 (DFS) on CHA‐SMU and 0.915 (OS) and 0.937 (DFS) on FAH‐SYSU, respectively, notably higher than either unimodal approach.

### 2.4 Incremental Value of CIOPM

Univariate and multivariate Cox analyses were performed on VC 1 and VC 2 to confirm the incremental utility of the CIOPM risk scores in NAT‐treated patients with breast cancer.

In VC 1, univariate analysis revealed that a high CIOPM_OS_ score (*p* < 0.001), higher pT stage (*p* = 0.043), negative pre‐NAT ER status (*p* = 0.042), negative pre‐NAT PR status (*p* = 0.044), pre‐NAT molecular subtypes of HR+HER2+ (*p* = 0.003) and HR−HER2− (*p* = 0.009), and no endocrine therapy (*p* = 0.035) were negatively associated with OS. The multivariate Cox analysis for OS in VC 1 demonstrated that the CIOPM_OS_ score remained an independent prognostic factor (*p* = 0.001; HR: 1.18 × 10^4^, 95% CI: 53.88–2.62 × 10^5^), along with the pT1 and pT2 stage (*p* = 0.018; HR: 0.01, 95% CI: 0.00–0.46) (Table S4). For DFS in VC 1, univariate analysis showed that a high CIOPM_DFS_ score (*p* < 0.001), high cT stage (*p* = 0.048), high cN stage (*p* = 0.001), high pT stage (*p* = 0.004), high pN stage (*p* < 0.001), pre‐NAT HER2 negativity (*p* = 0.041), the HR+HER2+ subtype (*p* = 0.013), and the presence of LVI after NAT (*p* < 0.001) were significant negative prognostic factors. In the multivariate analysis, only the CIOPM_DFS_ score retained significance (*p* < 0.001; HR: 1.27, 95% CI: 1.22–1.33) (Table S5).

In VC 2, univariate analysis for OS identified a high CIOPM_OS_ score (*p* < 0.001), high cT stage (*p* = 0.026), high pT stage (*p* = 0.003), negative pre‐NAT PR status (*p* = 0.014), the HR−/HER2− subtype (*p* < 0.001), and no endocrine therapy (*p* = 0.002) as factors negatively associated with survival. Multivariate analysis in VC 2 confirmed the independent prognostic value of the CIOPM_OS_ score (*p* < 0.001; HR: 147.96, 95% CI: 12.04–1.82 × 10^3^) and pT stage (*p* = 0.016) (Table S6). For DFS in VC 2, univariate analysis showed that a high CIOPM_DFS_ score (*p* < 0.001), high pT stage (*p* = 0.010), high pN stage (*p* = 0.013), and residual LVI after NAT (*p* = 0.009) were significant predictors of worse outcomes. The multivariate analysis demonstrated that patients with a high CIOPM_DFS_ risk score had a significantly higher risk of disease recurrence or death (*p* < 0.001; HR: 1.58 × 10^4^, 95% CI: 1.28 × 10^3^–1.96 × 10^5^), and pT3 and pT4 stage was also independently associated with DFS (*p* = 0.023; HR: 11.10, 95% CI: 0.01–0.71) (Table S7).

### 2.5 Subgroup Analysis of CIOPM Prognostic Performance

The prognostic stratification ability of CIOPM across distinct breast cancer subtypes in VC 1 and VC 2 was further evaluated. These results confirm that CIOPM maintains robust prognostic stratification and predictive accuracy across breast cancer molecular subtypes, particularly in HR+HER2−, HR−HER2−, and pCR subgroups, supporting its potential clinical utility in personalized risk assessment.

#### 2.5.1 Molecular Subtype

In VC 1, for OS, CIOPM effectively stratified patients into high‐risk and low‐risk subgroups within the HR+HER2− subtype (log‐rank *p* < 0.001; Figure S2A) and HR−HER2− subtype (log‐rank *p* < 0.001; Figure S2C). Significant differences in DFS were observed between risk subgroups in the HR−HER2+ subtype (log‐rank *p* < 0.001; Figure S2E). Time‐dependent ROC analysis in VC 1 demonstrated favorable predictive accuracy: in the HR+HER2− subtype, the 3‐year and 5‐year OS AUC values were 0.936 and 0.946, respectively (Figure S2B); in the HR−HER2− subtype, the corresponding AUC values were 0.917 and 0.897 (Figure S2D); and in the HR−HER2+ subtype, the 3‐year and 5‐year DFS AUC values both reached 1.000 (Figure S2F).

In VC 2, CIOPM did not achieve statistically significant prognostic stratification in the HR−HER2− subtype (OS, log‐rank *p* = 0.101; Figure S2G), while significant differences in DFS were observed in both the HR+HER2+ (log‐rank *p* < 0.001; Figure S2I) and HR−HER2 HR− (log‐rank *p* < 0.001; Figure S2K) subtypes. Time‐dependent ROC analysis in VC 2 revealed: in the HR−HER2− subtype, the 3‐year and 5‐year OS AUC values of 0.863 and 0.863 (Figure S2H); in the HR+HER2+ subtype, the 3‐year and 5‐year DFS AUC values of 1.000 and 0.972 (Figure S2J); and in the HR−HER2‐ subtype, the 3‐year and 5‐year DFS AUC values of 1.000 and 1.000 (Figure S2L).

#### 2.5.2 pCR and Non‐pCR Groups

In VC 1, CIOPM effectively stratified patients into high‐risk and low‐risk subgroups within the non‐pCR group for OS (log‐rank *p* < 0.001; Figure S3A). Significant differences in DFS were observed between risk subgroups in the non‐pCR group (log‐rank *p* < 0.001; Figure S2C). Time‐dependent ROC analysis in VC 1 demonstrated favorable predictive accuracy: in the non‐pCR group, the 3‐year and 5‐year OS AUC values were 0.957 and 0.944, respectively (Figure S3B), and the 3‐year and 5‐year DFS AUC values were 0.956 and 0.972, respectively (Figure S3D).

In VC 2, for OS, CIOPM effectively stratified patients into high‐risk and low‐risk subgroups within the non‐pCR group (log‐rank *p* < 0.001; Figure S3E). Significant differences in DFS were observed between risk subgroups in the non‐pCR group (log‐rank *p* < 0.001; Figure S2G). Time‐dependent ROC analysis in VC 1 demonstrated favorable predictive accuracy: in the non‐pCR group, the 3‐year and 5‐year OS AUC values were 0.920 and 0.953, respectively (Figure S3F), and the 3‐year and 5‐year DFS AUC values were 0.944 and 0.991, respectively (Figure S3H).

### 2.6 Comparison With the Foundation Models

We conducted comparative experiments with state‐of‐the‐art foundation models in digital pathology to assess the superiority of our proposed framework. Specifically, we compared our approach with CHIEF [18], a leading pathological image foundation model, and MUSK [19], a vision‐language model designed for multimodal integration.

As shown in Table 2, models based on CHIEF features achieved C‐indexes of 0.531 for OS and 0.504 for DFS on external VC 1 (CHA‐SMU), and 0.678 (OS) and 0.658 (DFS) on VC 2 (FAH‐SYSU). Using MUSK as the feature extractor yielded improved but still limited performance, with C‐indexes of 0.634 (OS) and 0.677 (DFS) on CHA‐SMU, and 0.818 (OS) and 0.789 (DFS) on FAH‐SYSU. Furthermore, we reimplemented MUSK's full multimodal pipeline, including its pretrained image and text encoders and original survival prediction head, fine‐tuned on our dataset using the same clinical variables. MUSK achieved C‐indexes of 0.818 (OS) and 0.789 (DFS) on FAH‐SYSU, which are substantially lower than our proposed model. These results indicate that the structured representation of clinical data via one‐hot encoding combined with our random survival forest fusion is more computationally efficient and more effective than general‐purpose vision‐language encoders trained on large‐scale pathology corpora that may not align well with our task or data distribution in our specific clinical context, where prognostic factors are discrete, well‐defined, and limited in number.

**TABLE 2:** The results of comparison with foundation models.

Models		C‐index (95% CI)			
		CIOPM	CHIEF	MUSK‐image only	MUSK‐multimodal data
VC 1					
(*n* = 142)	OS	0.933 (0.878–0.977)	0.531 (0.469–0.584)	0.437 (0.393–0.482)	0.634 (0.595–0.671)
	DFS	0.947 (0.895–0.983)	0.504 (0.429–0.561)	0.517 (0.473–0.562)	0.677 (0.645–0.709)
VC 2					
(*n* = 143)	OS	0.915 (0.850–0.960)	0.678 (0.878–0.977)	0.707 (0.663–0.752)	0.818 (0.785–0.841)
	DFS	0.937 (0.905–0.965)	0.658 (0.595–0.714)	0.683 (0.647–0.743)	0.789 (0.761–0.815)

Abbreviations: CI, confidence interval; CIOPM, ClinicHistomics Integrated Outcome Prediction Model; DFS, disease‐free survival; OS, overall survival; VC, validation cohort.

### 2.7 Comparison With Clinical Risk Assessment Models

As shown in Table 3, in VC 1, CIOPM demonstrated superior predictive performance for both OS and DFS compared to the RCB, AJCC, and Neo‐Bioscore systems. The C‐index for CIOPM in predicting OS was 0.933 (95% CI: 0.878–0.977), which was substantially higher than that of RCB (0.332), AJCC (0.369), and Neo‐Bioscore (0.365). Similarly, the C‐index for CIOPM in predicting DFS was 0.947 (95% CI: 0.895–0.983), which also exceeded the values for RCB (0.445), AJCC (0.466), and Neo‐Bioscore (0.433). In VC 2, CIOPM maintained superior predictive accuracy over AJCC (data for RCB and Neo‐Bioscore were not available in this cohort). The C‐index for CIOPM for OS was 0.915 (95% CI: 0.850–0.960), compared with 0.267 for AJCC, and for DFS, the C‐index was 0.937 (95% CI: 0.905–0.965), compared with 0.295 for AJCC.

**TABLE 3:** Comparison of CIOPM with RCB, AJCC, and Neo‐Bioscore.

Models		C‐index (95% CI)			
		CIOPM	RCB	AJCC	Neo‐Bioscore
VC 1 (*n* = 142)	OS	0.933 (0.878–0.977)	0.332 (0.190–0.475)	0.369 (0.235–0.503)	0.365 (0.235–0.494)
	DFS	0.947 (0.895–0.983)	0.445 (0.310–0.558)	0.466 (0.350–0.577)	0.433 (0.323–0.534)
VC 2 (*n* = 143)	OS	0.915 (0.850–0.960)	—	0.267 (0.143–0.422)	—
	DFS	0.937 (0.905–0.965)	—	0.295 (0.203–0.390)	—

Abbreviations: AJCC, the American Joint Committee on Cancer; DFS, disease‐free survival; OS, overall survival; RCB, residual cancer burden; VC, validation cohort.

### 2.8 Interpretability and Application of CIOPM

To investigate the importance of each feature in CIOPM for predicting OS and DFS in patients with NAT‐treated breast cancer, this study utilized a perturbation method to determine the importance of each feature in the model prediction process.

The top five predictive features of CIOPM for OS comprised WSI features, whether patients received endocrine therapy, pre‐NAT PR status, and LVI. The top five predictive features of CIOPM for DFS comprised WSI features, pre‐NAT PR status, maximum diameter of residual cancer in the lymph nodes, whether endocrine therapy was given, whether targeted therapy was given, and cTNM stage.

**FIGURE 3:**
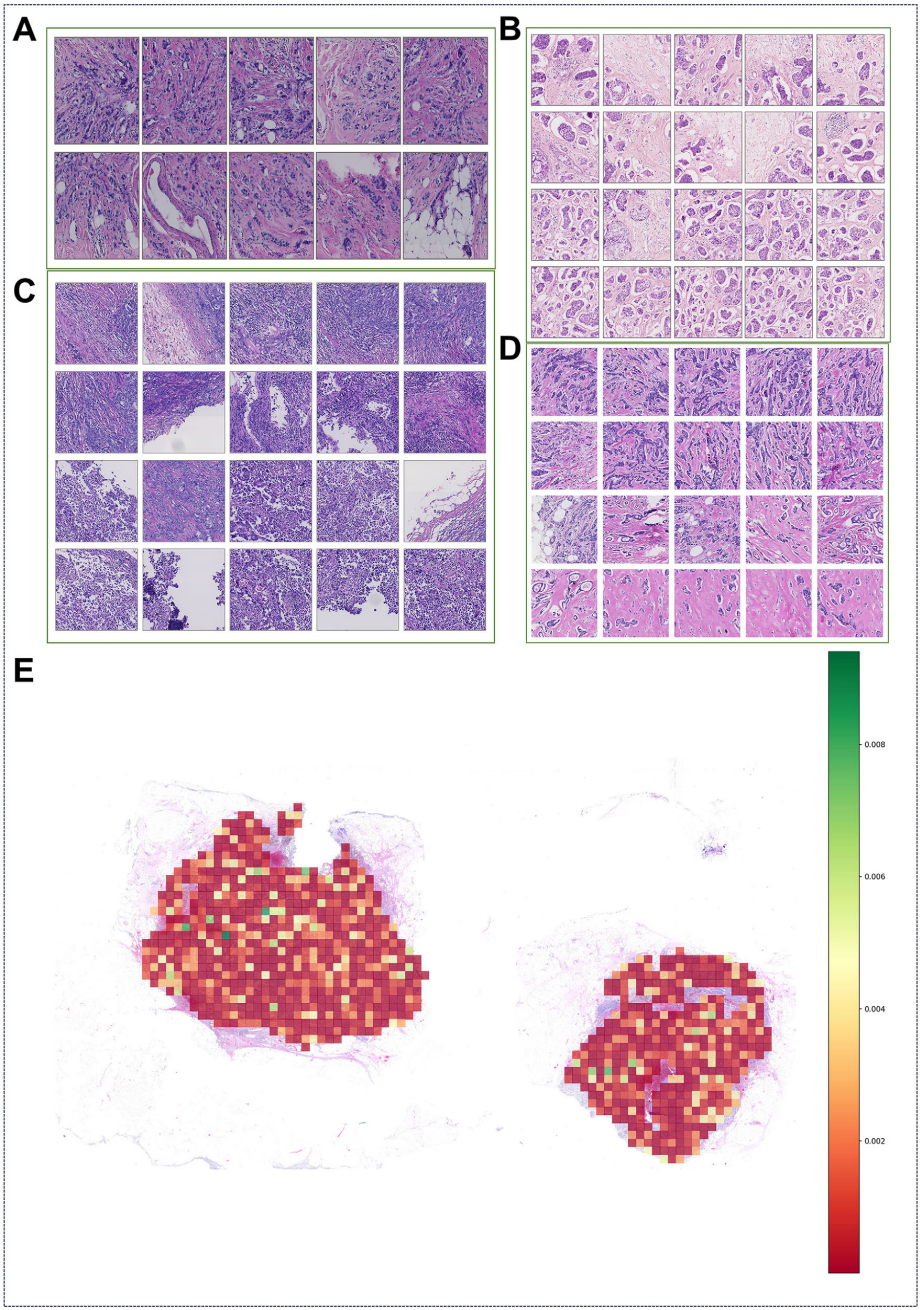
Four histomorphological features that contributed the most to the model: (A) fatty invasion, (B) tumor absorption area, (C) necrosis, and (D) more mature collagen fibers. (E) A typical example image of the contribution of necrosis to the model heatmap and a WSI heatmap based on the patch attention value. The patch has increasing attention values from left to right and from top to bottom, with the lowest in the upper‐left corner and the highest in the lower‐right corner. Green indicates a low attention value, while red denotes a high attention value.

Then, we obtained the attention value of each patch based on its contribution to CIOPM and generated a WSI‐level heatmap using ComplexHeatmap based on the patch attention value [20]. After reviewing the patches with the highest and lowest attention, histomorphological differences were identified and the impactful WSI features were investigated. By combining patch attention values into a WSI heatmap, we highlighted the following four traits that contributed most to the model: fatty invasion, tumor resorption, necrosis, and mature collagen fibers; patients exhibiting these features above may have a worse prognosis (Figure 3).

**FIGURE 4:**
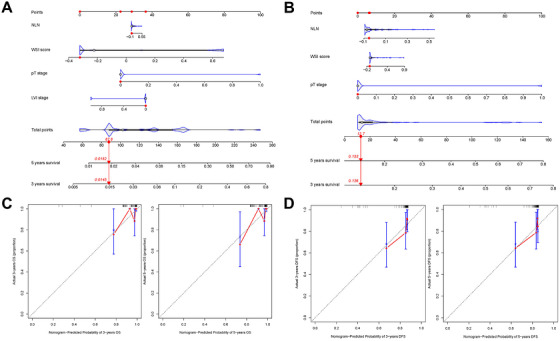
OS and DFS nomograms and calibration curves. Nomogram for OS (A) and DFS (B). The 3‐ and 5‐year calibration curves in (C) and (D) demonstrate good agreement between the predicted and actual OS and DFS values, respectively. The line segments in the nomogram that correspond to each variable are labeled with scales, and the length of the line segment represents the extent to which the factor contributes to the outcome of the event. LVI, vascular invasion: 0 for no invasion, 1 for invasion; NLN, the number of metastatic lymph nodes (standardized score); pT stage: 0 for pT0/pT1/pT2, 1 for pT3/pT4; WSI score, the whole‐slide image characteristic (standardized score). The error bars were defined as the standard error of the mean, which represents the 95% CI.

WSI features, pT stage, and post‐NAT LVI status were important OS nomogram indicators. WSI features, pT stage, and the number of metastatic lymph nodes were important DFS nomogram indicators. In VC 1, and VC 2, the OS nomogram C‐indexes (95% CIs) were 0.695 (0.52–0.87), and 0.895 (0.80–0.99), respectively; the DFS nomogram C‐indexes were 0.729 (0.61–0.85) and 0.538 (0.27–0.81), respectively (Figure 4).

## 3 Discussion

In this retrospective, multicenter study, we developed and validated an AI integrated model to predict the outcomes of NAT‐treated patients with breast cancer using the complex and variable postoperative pathological images and comprehensive pre‐ and post‐clinicopathological features. In addition, we extracted some significant prognostic factors that had not been previously noted from the WSIs.

Pathological WSIs serve as the gold standard for cancer diagnosis and are promising prognostic indicators for NAT‐treated patients with breast cancer. An accurate prognosis can provide an excellent reference for identifying postoperative adjuvant treatments following NAT. However, some current prognostic systems for NAT‐treated patients with breast cancer have limitations, such as insufficient predictive power and feasibility. In practice, increasing the number and complexity of prognostic biomarkers prolongs decision‐making time and costs, while evaluation relies on invasive tissue sampling. The diagnosis of breast cancer always depends on histopathological morphology, which is also a crucial prognostic factor. However, WSIs contain unexplored information on disease mechanisms and progression. Recently, DL has shown advantages in medical image analysis. DL‐based histological scores better predict NAT response than common characteristics, demonstrating the potential for precise patient categorization [17]. Thus, WSIs may provide incremental value for NAT prognosis predictors. Despite ongoing research on predicting the NAT response in patients with breast cancer, few studies have examined outcome prediction, due to the difficulty in incorporating WSIs and the complete follow‐up data needed for multimodal prognostic prediction. Some studies have shown that radiomic signatures help select patients who may benefit from adjuvant chemotherapy by predicting distant metastasis in rectal cancer patients [21]. Our findings suggest that DL algorithms can obtain prognostic image features reflecting disease mechanisms, helping improve prognostic prediction models for NAT‐treated patients with breast cancer.

Multimodal models using several‐dimensional features, including transcriptomic, metabolomic, epigenetic, and proteomic data, have been widely applied in bioinformatics for multiomics data processing. Studies on the NAT response and prognosis of NAT‐treated breast cancer patients involve various markers, such as clinicopathological, molecular, and WSI features [8]. However, most current studies on post‐NAT residual cancer have constructed predictive models using single‐dimensional features, failing to adequately integrate multidimensional patient information [22, 23, 24]. A recent study published in *Nature* validated the advantages of multimodal models for pCR prediction in NAT‐treated breast cancer patients [9]. Similarly, our results showed that the multimodal model combining image and clinicopathological features had superior predictive efficacy compared to the single WSI modality model. Preliminary interpretability analysis for the DL model revealed four key imaging features (fat infiltration, tumor resorption, necrosis, and increased mature collagen fibers) with potential prognostic relevance in bireast cancer. These findings could provide useful information for prognostic assessment and individualized breast cancer treatment.

The model's strong predictive performance for OS and DFS underscores its clinical utility. A more accurate prognostic risk score could aid doctors and patients in selecting personalized, effective treatment strategies. Furthermore, our model could stratify patients into multiple subgroups, suggesting that prognostic predictive risk scores could potentially help guide risk stratification across various subgroups. Therefore, CIOPM in our study, based on clinicopathological and WSI features, may be a useful tool for physicians. Nomograms based on clinicopathological factors and pathological WSIs were also constructed as easy‐to‐use visualization tools. Users do not need to participate in manual data processing or model operation; basic clinical research knowledge is sufficient for model application. The nomogram calibration curves showed good agreement between the predicted and actual outcomes. Our findings suggest that nomograms could be used to convert complex prediction models into understandable graphs to improve the assessment of recurrence and death risks in patients with breast cancer treated with NAT.

To assess the added value of our multimodal approach, we compared CIOPM with recent foundation models in digital pathology, including CHIEF [18] and MUSK [19]. Although these models reduce annotation burden through unsupervised feature extraction, their applicability to post‐neoadjuvant breast cancer prognosis, where treatment‐induced histopathological alterations introduce extensive morphological noise (e.g., cytoplasmic vacuolation, bizarre giant cells, stromal fibrosis), remains uncertain, as such noise may distract feature extractors from prognostically critical regions. Our experiments confirmed this: models using CHIEF‐derived features achieved C‐index values of only 0.678 (OS) and 0.658 (DFS) on external cohorts, substantially lower than ours, and while MUSK performed better (up to 0.818 and 0.789), it still underperformed relative to our approach. These results suggest that in this nuanced context, supervised learning on pathologist‐defined regions aligns more closely with diagnostic gold standards. Furthermore, comparing our structured clinical encoding (one‐hot representation with random survival forest) with MUSK's vision‐language pipeline revealed its inferior performance, indicating that for well‐defined, limited clinical variables, structured encoding with ensemble methods is more effective than general‐purpose models trained on diverse corpora. Collectively, these findings confirmed that our manual annotation strategy and multimodal fusion framework remain necessary and effective for accurate prognosis prediction in this challenging clinical scenario.

We identified previously unrecognized WSI characteristics related to prognosis by interpreting the image features, providing crucial clinical insights and novel ideas for DL model interpretation. Moreover, our prediction model uses H&E images and clinicopathological data stored from diagnosis, without incurring additional healthcare costs. Predicting patients’ prognosis via ML algorithms based on acquired NAT‐treated breast cancer images avoids invasive patient examination. The continuous accumulation of numerous NAT‐treated breast cancers in the TC can improve the model's predictive efficacy, potentially exceeding that of less experienced physicians.

The current study has several limitations that should be considered. First, the sample size was limited, and data were retrospectively collected. Larger, multicenter, well‐designed prospective studies may be needed to further validate the efficacy of prediction. Second, although clinicopathological and imaging features provide a relatively comprehensive set of patient data, incorporating genetic factors such as genetic information could improve prognostic prediction performance.

## 4 Materials and Methods

### 4.1 Study Design and Participants

The inclusion criteria included: (1) patients diagnosed with primary invasive breast carcinoma of no special type without distant metastasis; (2) patients receiving NAT regimens with anthracycline, taxane, or both (≥ 4 cycles) and no prior therapy; (3) HER2+ patients receiving targeted therapy; and (4) patients undergoing surgery after NAT. The exclusion criteria were: (1) patients lacking NAT regimen or cycle information; (2) patients receiving non‐standard regimens; and (3) patients with inflammatory, recurrent, bilateral, or multifocal disease. The strategies for dividing the dataset can be found in Supporting Information.

Postoperative H&E slides were collected from four hospitals. Representative sections (1, 2, 3 slides) were selected based on morphological assessment, with the criteria being maximal histological heterogeneity for heterogeneous tumors, maximal residual tumor for non‐pCR cases, and maximal tumor bed for pCR cases. Then, the collected WSIs were digitized at 40× magnification using a NanoZoomer XR scanner (Hamamatsu). The following data were collected from the databases: age, sex, clinical stage, hormone receptor status, HER2 status, Ki67 status, nuclear grade, LVI status, Nottingham grade, treatments, and pathologic stage. Details of the parameter evaluation can be found in Supporting Information. OS was defined as the time from surgery to death from any cause. Patients without an event were excluded at the last follow‐up date. The DFS was defined as the time from enrollment or curative surgery to the first occurrence of distant metastasis. Patients without events were excluded at the last follow‐up.

### 4.2 Model Development

All available clinical variables were initially included. Pre‐selection was performed before model construction by excluding variables with high missing rates and strong collinearity. For pathological image data, a professional pathologist delineated the cancerous area of each image to remove irrelevant information. Guided by two pathologists with a long years of diagnostic pathology experience, we selected and labeled the region of interest (ROI). In this study, we used the Automated Slide Analysis Platform (ASAP) 1.9.0 software tool to label residual tumors and tumor beds after NAT. The ASAP user interface is illustrated in Figure S4. The following criteria were used for ROI selection and labeling: the tumor bed area was outlined for cases without residual cancer after NAT (Figure S5). For cases with residual cancer after NAT, all residual cancers were outlined as much as possible. Maximal sampling was performed for highly heterogeneous tumors (Figure S6). Due to the large image sizes, traditional convolutional neural networks cannot handle these images directly. For each patient, we grouped the 1024 × 1024‐pixel images created from the cancerous regions into a single bag. We extracted features from the pathological images using EfficientNet‐B7 pretrained on ImageNet to further reduce computational complexity.

Furthermore, we gathered the baseline characteristics of the patients, such as age and laterality. Missing values were imputed with Not‐a‐Number (NaN) for the category attribute data in the table and then encoded using one‐hot encoding. For numerical data, missing values were imputed with the mean of the corresponding attribute, and features were normalized by scaling to unit variance.

**FIGURE 5:**
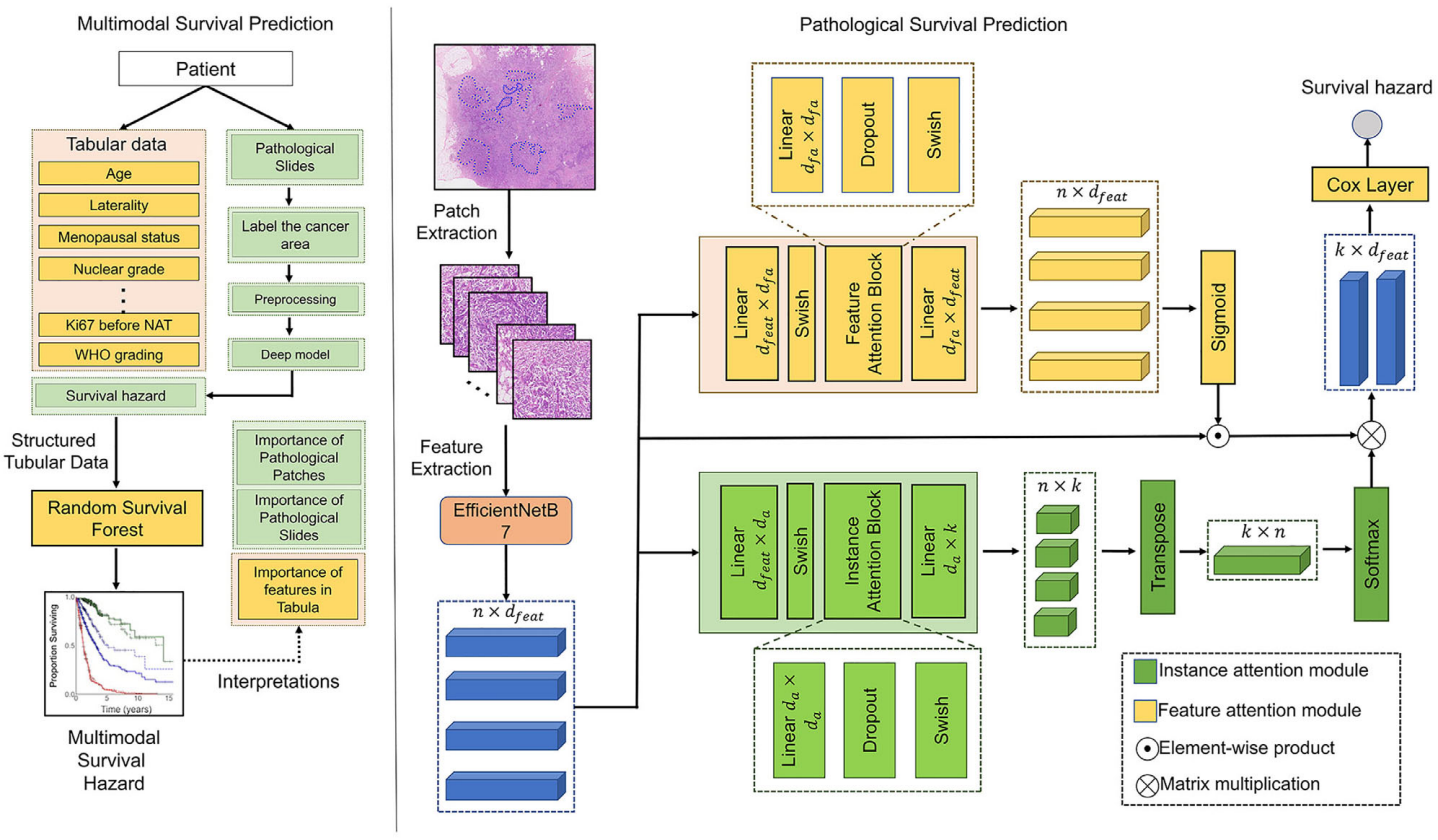
Construction of a multimodal model for predicting prognosis in patients with breast cancer receiving neoadjuvant therapy (NAT). The left section depicts the unimodal model based on clinicopathological data, where patient tabular features (e.g., age, menopausal status, nuclear grade, and Ki67) are used to predict survival hazard. The right section illustrates the unimodal model based on whole‐slide images (WSIs): after tumor region annotation and preprocessing, deep features are extracted and weighted by feature and instance attention modules to generate a WSI‐derived risk score. The multimodal model is built by incorporating this WSI risk score as an additional feature into the clinicopathological data, followed by a Cox layer to produce the final survival prediction.

We developed an attention‐based multiple‐instance learning method for survival modeling [25]. A pathological image X can be seen as a bag of n instances, such as $X=\{x_{1},\text{…},x_{n}\}$, where each instance was a patch in the image. Let $Z=\{z_{1},\text{…},z_{n}\}$ be a bag of n embeddings corresponding to $X=\{x_{1},\text{…},x_{n}\}$. We employ an attention‐based fusion strategy to fuse the n different instance embeddings as shown in Figure 5. A feature attention module was used to retain the most informative features. Meanwhile, an instance attention module fused different instances based on learnable attention weights. Finally, we used a linear neural network to predict survival. The Cox proportional hazards model was adopted for estimating the hazard function [26]. The corresponding loss function can be written as Equation (1), where U is the set of uncensored patients and $R_{i}$ is the set of patients whose survival time is longer than that of the patient i.

(1) $$L_{cox}=-\sum_{i\in U}\left(p_{i}-\log\sum_{j\subset R_{i}}e^{p_{j}}\right)$$

The model was optimized by gradient descent using the AdamW optimizer [27]. The hyperparameters, including the learning rate, layer size, and model depth, were searched, and selected using the validation dataset. No model updating or recalibration was performed following the model evaluation, neither for the overall cohort nor for any specific sociodemographic groups or settings. No class imbalance methods were used. In this study, no specific algorithmic approaches or data mitigation strategies were used to explicitly address model fairness.

### 4.3 Model Validation

No formal sample size calculation was performed before model construction. The sample size was determined according to the widely accepted events per variable ≥ 10 criterion for prediction model development. The total number of events and participants in both the development and validation sets met the methodological requirements and was adequate for model construction and evaluation. We concatenated the prediction results from the pathological modality as a new attribute into the original tabular data to integrate information from the pathological and tabular modalities. We then employed random survival forests implemented in the scikit‐survival package for multimodal survival modeling [28, 29, 30]. Furthermore, to validate the superiority of our multimodal model, we independently conducted ablation experiments using the clinicopathological text modality and the pathology image modality. To evaluate the predictive performance of our model, we compared it with existing foundation models, including CHIEF [18] and MUSK [19].

### 4.4 Model Interpretation

For pathological modality survival prediction, the learned attention value indicates the importance of different patches. Patches with greater attention values were considered more important in the final model predictions.

We employed a permutation‐based method in the eli5 package to interpret the random survival forests. Feature importance was computed by measuring how model performance decreased when a feature was permuted.

### 4.5 Incremental Value of CIOPM

We developed OS and DFS nomograms based on the major parameters affecting the model to evaluate the contribution of certain metrics to the model. We employed a permutation‐based method in the eli5 package to interpret the random survival forests [31]. Specifically, the feature importance was computed by measuring how the model's performance decreased when a feature was permuted.

The clinical features that were statistically significant according to univariate Cox analysis in TC were selected. A Lasso–Cox model was built in the training cohort using fivefold cross‐validation, and standardized data were used to select clinicopathological indicators and image features with non‐zero regression coefficients as variables for the nomograms. These clinical markers were used to develop OS and DFS nomograms.

Nomogram models were validated on both the primary and validation cohorts. The formulas for the nomograms are as follows:

OS nomogram = −0.8666 × LVI + 2.2047 × pT + 2.2660 × WSI score + 0.2606 × NLN
DFS nomogram = 1.5842 × pT + 0.2943 × WSI score + 0.9384 × NLN

 where OS is overall survival, DFS is disease‐free survival, LVI is lymphovascular invasion, pT is pathologic tumor size, WSI is the whole‐slide image predicted risk score, and NLN is the number of positive lymph nodes.

### 4.6 Statistics and Reproducibility

The C‐index and the area under the ROC curve (AUC) were used to assess the prediction performance of each model in the two validation cohorts. Potential associations between the multimodal models and OS and DFS were initially assessed in the cohorts. Kaplan–Meier curve thresholds were determined using the surv_cutpoint function (R package survminer), which identifies the optimal cutoff by maximizing the log‑rank statistic. For each dataset, the function selects the risk score value that best separates survival outcomes between high‑ and low‑risk groups, with a minimum subgroup proportion of 10% enforced to avoid extreme splits. Thus, the cut‑point is dataset‑specific rather than fixed, enabling data‑driven and clinically meaningful stratification across cohorts [32]. The ROC curve was used to assess the prognostic accuracy of the OS and DFS multimodal models for patient stratification in the primary and validation cohorts. The ROC curves for 3‐ and 5‐year OS and DFS were plotted with AUC values calculated for all cohorts. Kaplan–Meier analysis was used to the outcomes of different groups. Nomogram performance was evaluated using the C‐index, calibration curves, and decision curve analysis. Univariate and multivariate Cox analyses were used to evaluate the associations between the model‐predicted risk score and prognosis. For continuous variables, *t*‐tests or Mann–Whitney *U* tests were used, while for categorical variables, chi‐squared or Fisher's tests were used as appropriate. A significant difference between groups was defined as a two‐sided *p* value less than 0.05.

The DL component consists of five instance attention modules (each with a network dimension of 256) and five feature attention modules (each with a network dimension of 128). The Top‑K patch selection strategy employs *K* = 3. We used 22 decision trees, each with a maximum depth of 96, for the random forest model. The minimum number of samples required to split an internal node is 8, and each leaf node must contain at least 6 samples.

Statistical analyses were performed using Rstudio (version 4.1.3) and SPSS (version 26.0). GraphPad Prism 9.5.1 was used to generate ROC curves, Kaplan–Meier, Nomogram, and calibration curves.

## 5 Conclusion

In conclusion, CIOPM integrates histomorphological and clinical data to accurately predict prognosis in NAT‐treated breast cancer, outperforming conventional models and supporting personalized adjuvant therapy.

## Author Contributions

Y.W., W.L., Z.H., J.Y., and H.B. designed the study. Y.W., W.L., Z.H., F.Y., F.L., B.W., J.Y., and H.B. managed the project. Y.W., F.L., Y.Z., H.L., H.S., A.H., and X.X. prepared the cases. Y.W., F.L., H.L., B.W., and Y.Z. reviewed the slides and evaluated clinicopathological parameters. Y.W., W.L., Y.Y., Z.H., and H.L. analyzed and interpreted the data. Y.W., W.L., and Z.H. wrote the manuscript, which was reviewed by all co‐authors. All authors contributed to the writing of the manuscript and approved the final version for submission and publication.

## Ethics Statement

This retrospective multicenter study followed the Declaration of Helsinki. This study was approved by the Ethics Committee of West China Hospital, Sichuan University (No.764/2021). All procedures conformed to the established protocol, and informed consent was obtained from all participants.

## Conflicts of Interest

Author Zongbo Han was an employee of Tencent but has no potential relevant financial or non‐financial interests to disclose. The other authors declare no conflicts of interest.

## Supporting information




**Figure S1** The distribution of the number of 1024×1024 patches at 40× magnification.
**Figure S2** Prognostic stratification and predictive performance of CIOPM in breast cancer molecular subtypes of validation cohorts.
**Figure S3** Prognostic stratification and predictive performance of CIOPM in breast cancer non‐pCR of validation cohorts.
**Figure S4** The working interface of ASAP 1.9.0 software and the tumor area labelling.
**Figure S5** The labelling process for tumor beds.
**Figure S6** The labelling process for heterogeneous tumors.
**Table S1** The results of the ablation study.
**Table S4** Summary of univariate and multivariate Cox model estimates for OS (CIOPM) on VC 1.
**Table S5** Summary of univariate and multivariate Cox model estimates for DFS (CIOPM) on VC 1.
**Table S6** Summary of univariate and multivariate Cox model estimates for OS (CIOPM) on VC2.
**Table S7** Summary of univariate and multivariate Cox model estimates for DFS (CIOPM) on VC2.
**Table S8** The details of the antibodies.
**Table S9** Point assignments for the yAJCC, RCB, and Neo‐Bioscore Staging Systems.

**Table S2** The corresponding risk values for each case predicted by CIOPM_OS_ (Table S2A) and CIOPM_DFS_ (Table S2B) on validation cohort 1.

**Table S3** The corresponding risk values for each case predicted by CIOPM_OS_ (Table S3A) and CIOPM_DFS_ (Table S3B) on validation cohort 2.

## Data Availability

The dataset supporting the conclusions of this article is available upon request with the institutions’ policies. All requests for access to in‐house data will be addressed to the corresponding authors, Jianhua Yao (jianhuayao@tencent.com) or Hong Bu (hongbu@scu.edu.cn), and will be processed in accordance with the institutional guidelines of West China Hospital, Sichuan University. A material‐transfer or data‐usage agreement will be required between West China Hospital and the receiving organization. The requesting organization must provide comprehensive details, including the name and full contact information of the individual and institution making the request, along with specific identification of the data being requested. In addition, the requesting organization must clearly state the intended purpose of the data transfer and provide assurances that the transferred data will only be used for non‐commercial academic and educational purposes in compliance. All model training codes have been made available in the GitHub repository: https://github.com/TencentAILabHealthcare/MultimodalSurvivalAnalysis.
