# Supplementary material for: Development and Validation of a Pathomics Model for Prognosis Prediction in Neoadjuvant Therapy‐Treated Breast Cancer: A Retrospective, Multicenter Study
Source: MedComm (2020). 2026 Jul 2;7(7):e70826. doi: 10.1002/mco2.70826 (PMC13329266; doi:10.1002/mco2.70826)
Supplement: Supplementary file 1 — Figure S1 The distribution of the number of 1024×1024 patches at 40× magnification. Figure S2 Prognostic stratification and predictive performance of CIOPM in breast cancer molecular subtypes of validation cohorts. Figure S3 Prognostic stratification and predictive performance of CIOPM in breast cancer non‐pCR of validation cohorts. Figure S4 The working interface of ASAP 1.9.0 software and the tumor area labelling. Figure S5 The labelling process for tumor beds. Figure S6 The labelling process for heterogeneous tumors. Table S1 The results of the ablation study. Table S4 Summary of univariate and multivariate Cox model estimates for OS (CIOPM) on VC 1. Table S5 Summary of univariate and multivariate Cox model estimates for DFS (CIOPM) on VC 1. Table S6 Summary of univariate and multivariate Cox model estimates for OS (CIOPM) on VC2. Table S7 Summary of univariate and multivariate Cox model estimates for DFS (CIOPM) on VC2. Table S8 The details of the antibodies. Table S9 Point assignments for the yAJCC, RCB, and Neo‐Bioscore Staging Systems. [file MCO2-7-e70826-s003.docx]

**Development and validation of a pathomics model for prognosis prediction in neoadjuvant therapy-treated breast cancer: a retrospective, multicenter study**

Yani Wei^1,2,3#^, Wei Lou^4#^, Zongbo Han^5,6^, Fan Yang^6^, Fengling Li^1,2^, Haofeng Li^7^, Huijuan Shi^3^, Bing Wei^1^, Hongjun Li^3^, Yuanyuan Zhao^8^, Xiuli Xiao^9^, Yongquan Yang^2^, Anjia Han^3^, Jianhua Yao^6*^, Hong Bu^1,2*^

^1^Department of Pathology, West China Hospital, Sichuan University, Chengdu, Sichuan, China. ^2^Institute of Clinical Pathology, West China Hospital, Sichuan University, Chengdu, Sichuan, China. ^3^Department of Pathology, the First Affiliated Hospital, Sun Yat-sen University, Guangzhou, 510080, China. ^4^College of Mathematical Medicine, Zhejiang Normal University, Jinhua, Zhejiang, China. ^5^State Key Laboratory of Networking and Switching Technology, Beijing University of Posts and Telecommunications. ^6^Tencent AI Lab, Shenzhen, Guangdong, China. ^7^School of Systems Science and Engineering, Sun Yat-sen University, Guangzhou, 510275, China. ^8^Department of Pathology, Shanxi Province Cancer Hospital/Shanxi Hospital Affiliated to Cancer Hospital, Chinese Academy of Medical Sciences/Cancer Hospital Affiliated to Shanxi Medical University, Taiyuan, China. ^9^Department of Pathology, The Affiliated Hospital of Southwest Medical University, Luzhou, Sichuan, China. ^#^These authors contributed equally: Yani Wei and Wei Lou.

***Correspondence to:**

Jianhua Yao, Tencent AI Lab, Shenzhen, Guangdong, China; E-mail: [jianhuayao@tencent.com](mailto:jianhuayao@tencent.com).

Hong Bu, West China Hospital, Sichuan University, Chengdu, Sichuan, China; E-mail: [hongbu@scu.edu.cn](mailto:hongbu@scu.edu.cn).

**Running title:** Outcome prediction for breast cancer

**Keywords**: breast cancer, whole slide image, neoadjuvant therapy, prognosis, deep learning

**Supplementary Methods**

**Dataset dividing**

The dataset in this study was divided according to the following strategies: (1) grouping by occurrence of events, 1) categorizing patients into NAT breast cancer cohorts with or without mortality, and 2) categorizing patients into NAT breast cancer cohorts with or without distant metastasis, and (2) the cases were stratified and sorted from the smallest to the largest according to overall survival (OS) and disease free survival (DFS) during follow-up. The data used for the OS and DFS prediction models were labeled with both event occurrence and survival time.

**Parameter evaluation**

Parameters were independently evaluated by two pathologists, and disagreements were resolved by a third pathologist. The antibodies used for immunohistochemical staining of ER, PR, HER2, and Ki67 are listed in Table S8. The American Society of Clinical Oncology/College of American Pathologists (ASCO/CAP) guideline criteria were used to evaluate ER, PR, and HER2 expression^1,2^. Ki67 expression was evaluated using a cut-off value of 20%^3^. Patients were classified into four subtypes based on ER, PR, and HER2 status: HR+HER2-, HR+HER2+, HR-HER2+, and HR-HER2-. Post-NAT sTILs were evaluated on hematoxylin and eosin (H&E)-stained slides using the international recommended guidelines^4^, with a range of 1% to 90%, and were classified as low (<20%) or high (≥20%). According to the 10% threshold, post-NAT iTILs were classified as low (<10%) or high (≥10%). Post-NAT LVI was defined as lymphovascular invasion of cancer cells. pCR was defined as the absence of residual invasive carcinoma in both the primary breast site and axillary lymph nodes (ypT0/is and ypN0)^5^.

We also evaluated the performance of the yAJCC^6^, residual cancer burden (RCB)^7^, and Neo-Bioscore staging systems^8^. Table S9 provides comprehensive information on these systems. DFS was defined as the time from surgery to death, local recurrence, or distant recurrence, whichever came first. OS was defined as the time from the operation to death.

**References**

1. Hammond ME, Hayes DF, Dowsett M, et al. American Society of Clinical Oncology/College Of American Pathologists guideline recommendations for immunohistochemical testing of estrogen and progesterone receptors in breast cancer. *Journal of clinical oncology : official journal of the American Society of Clinical Oncology*. 2010;28(16):2784-95.

2. Wolff AC, Hammond MEH, Allison KH, et al. Human Epidermal Growth Factor Receptor 2 Testing in Breast Cancer: American Society of Clinical Oncology/College of American Pathologists Clinical Practice Guideline Focused Update. *Journal of clinical oncology : official journal of the American Society of Clinical Oncology*. 2018;36(20):2105-2122.

3. Goldhirsch A, Winer EP, Coates AS, et al. Personalizing the treatment of women with early breast cancer: highlights of the St Gallen International Expert Consensus on the Primary Therapy of Early Breast Cancer 2013. *Annals of oncology : official journal of the European Society for Medical Oncology*. 2013;24(9):2206-23.

4. Salgado R, Denkert C, Demaria S, et al. The evaluation of tumor-infiltrating lymphocytes (TILs) in breast cancer: recommendations by an International TILs Working Group 2014. *Annals of oncology : official journal of the European Society for Medical Oncology*. 2015;26(2):259-71.

5. Cortazar P, Zhang L, Untch M, et al. Pathological complete response and long-term clinical benefit in breast cancer: the CTNeoBC pooled analysis. *Lancet (London, England)*. 2014;384(9938):164-72.

6. Giuliano AE, Edge SB, Hortobagyi GN. Eighth Edition of the AJCC Cancer Staging Manual: Breast Cancer. *Annals of surgical oncology*. 2018;25(7):1783-1785.

7. Symmans WF, Peintinger F, Hatzis C, et al. Measurement of residual breast cancer burden to predict survival after neoadjuvant chemotherapy. *Journal of clinical oncology : official journal of the American Society of Clinical Oncology*. 2007;25(28):4414-22.

8. Mittendorf EA, Vila J, Tucker SL, et al. The Neo-Bioscore Update for Staging Breast Cancer Treated With Neoadjuvant Chemotherapy: Incorporation of Prognostic Biologic Factors Into Staging After Treatment. *JAMA oncology*. 2016;2(7):929-36.

**Figures**

**Figure S1** The distribution of the number of 1024×1024 patches at 40× magnification.

**Figure S2** Prognostic stratification and predictive performance of CIOPM in breast cancer molecular subtypes of validation cohorts.

**Figure S3** Prognostic stratification and predictive performance of CIOPM in breast cancer non-pCR of validation cohorts.

**Figure S4** The working interface of ASAP 1.9.0 software and the tumor area labelling.

**Figure S5** The labelling process for tumor beds.

**Figure S6** The labelling process for heterogeneous tumors.

**Tables**

**Table S1** The results of the ablation study.

**Table S2** The corresponding risk values for each case predicted by CIOPM_OS_ (Table S2A) and CIOPM_DFS_ (Table S2B) on validation cohort 1.

**Table S3** The corresponding risk values for each case predicted by CIOPM_OS_ (Table S3A) and CIOPM_DFS_ (Table S3B) on validation cohort 2.

**Table S4** Summary of univariate and multivariate Cox model estimates for OS (CIOPM) on VC 1.

**Table S5** Summary of univariate and multivariate Cox model estimates for DFS (CIOPM) on VC 1.

**Table S6** Summary of univariate and multivariate Cox model estimates for OS (CIOPM) on VC2.

**Table S7** Summary of univariate and multivariate Cox model estimates for DFS (CIOPM) on VC2.

**Table S8** The details of the antibodies.

**Table S9** Point assignments for the yAJCC, RCB, and Neo-Bioscore Staging Systems.


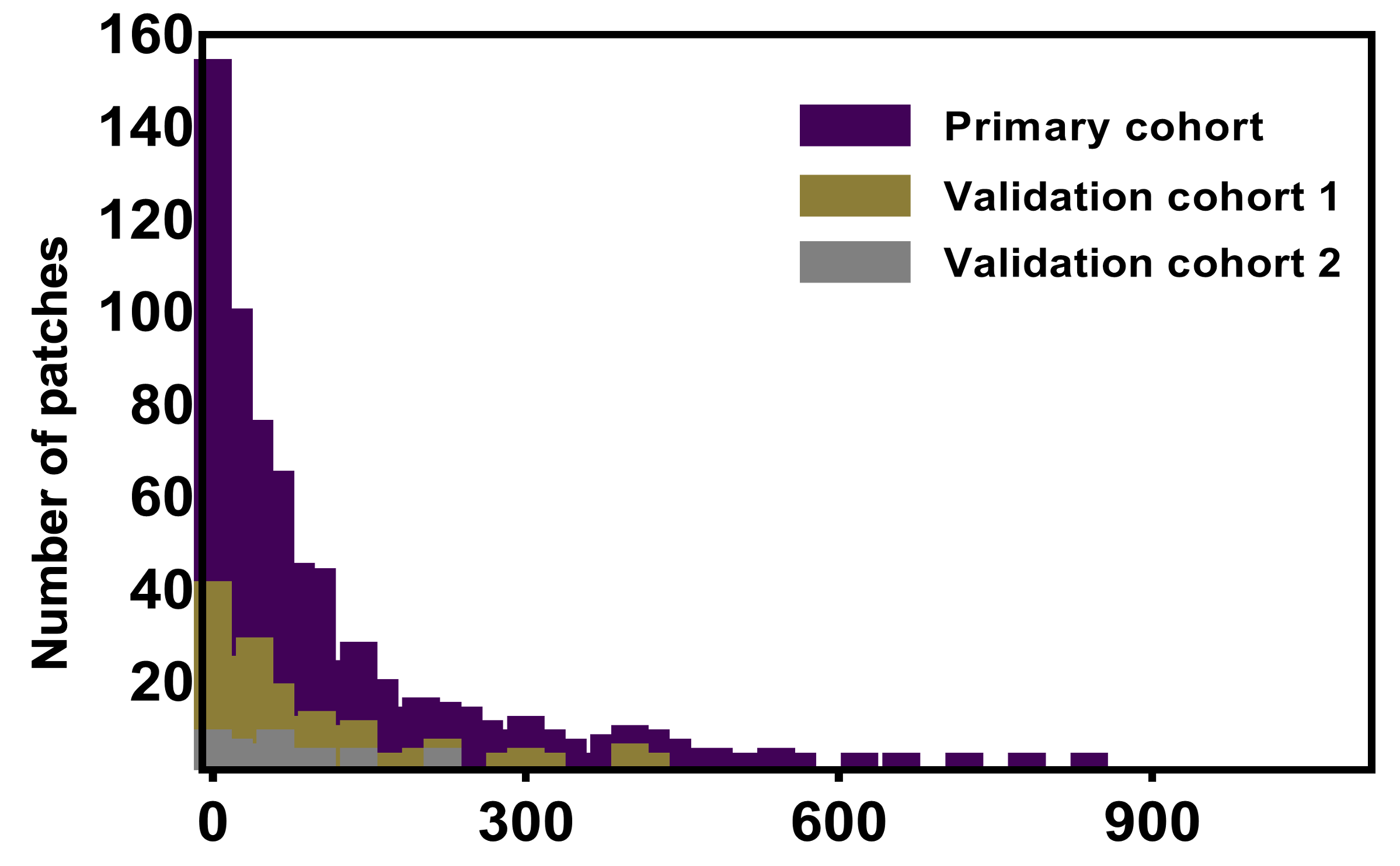


**FIGURE S1.** The distribution of the number of 1024×1024 patches at 40× magnification.


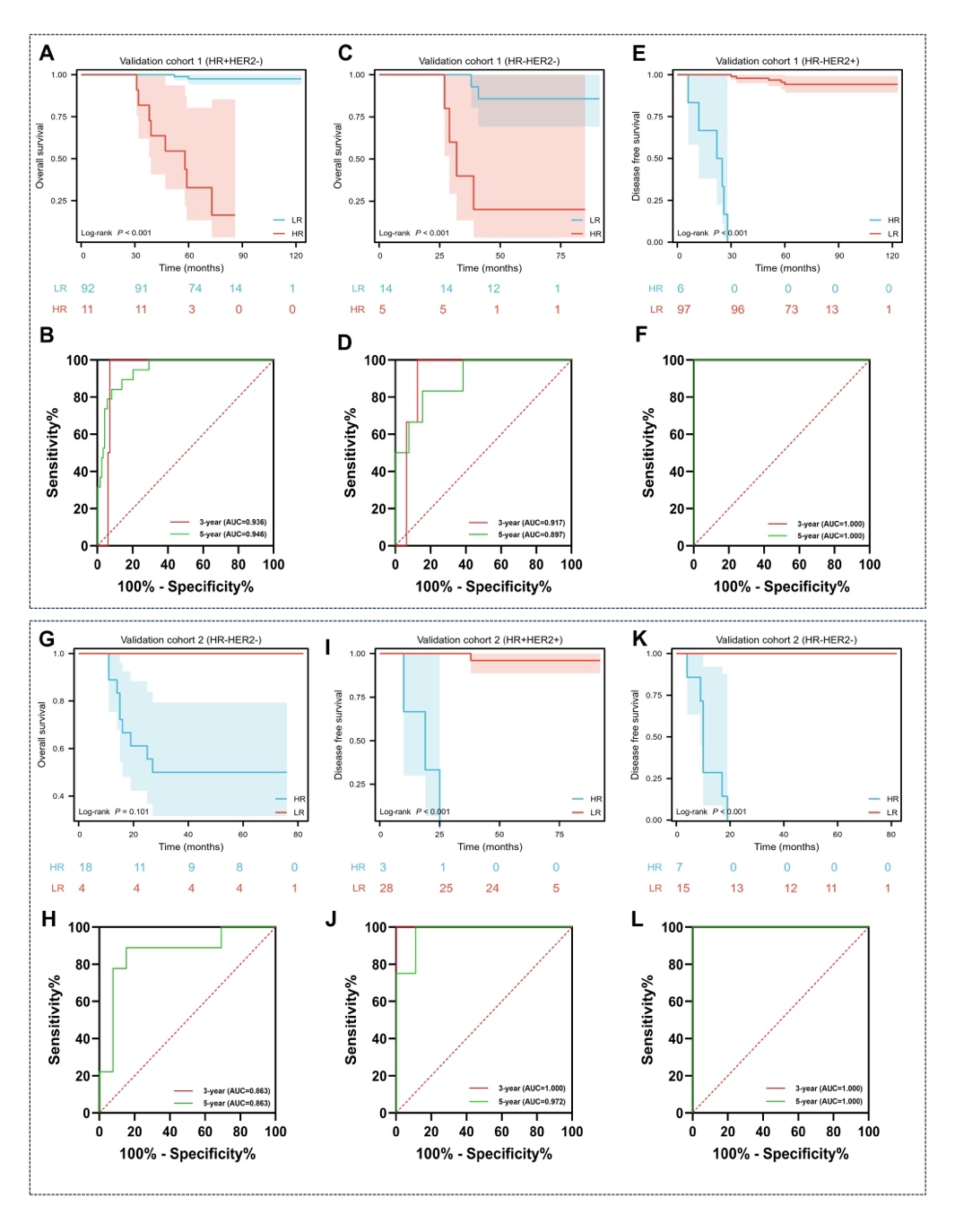


**FIGURE S2.** Prognostic stratification and predictive performance of CIOPM in breast cancer molecular subtypes of validation cohorts. VC 1: (A) Kaplan–Meier curves for OS (HR+HER2- subtype); (B) Time-dependent ROC curves for 3/5-year OS prediction (HR+HER2- subtype); (C) OS Kaplan–Meier curves (HR-HER2- subtype); (D) 3/5-year OS time-dependent ROC curves (HR-HER2- subtype); (E) DFS Kaplan–Meier curves (HR-HER2+ subtype); (F) 3/5-year DFS time-dependent ROC curves (HR-HER2+ subtype). VC 2: (G) OS Kaplan–Meier curves (HR-HER2- subtype); (H) 3/5-year OS time-dependent ROC curves (HR-HER2- subtype); (I) DFS Kaplan–Meier curves (HR+HER2+ subtype); (J) 3/5-year DFS time-dependent ROC curves (HR+HER2+ subtype); (K) DFS Kaplan–Meier curves (HR-HER2- subtype); (L) 3/5-year DFS time-dependent ROC curves (HR-HER2- subtype). Abbreviations: CIOPM, ClinicHistomics Integrated Outcome Prediction Model; OS, overall survival; DFS, disease-free survival; VC, Validation Cohort; ROC, receiver operating characteristic; HER2, human epidermal growth factor receptor 2.


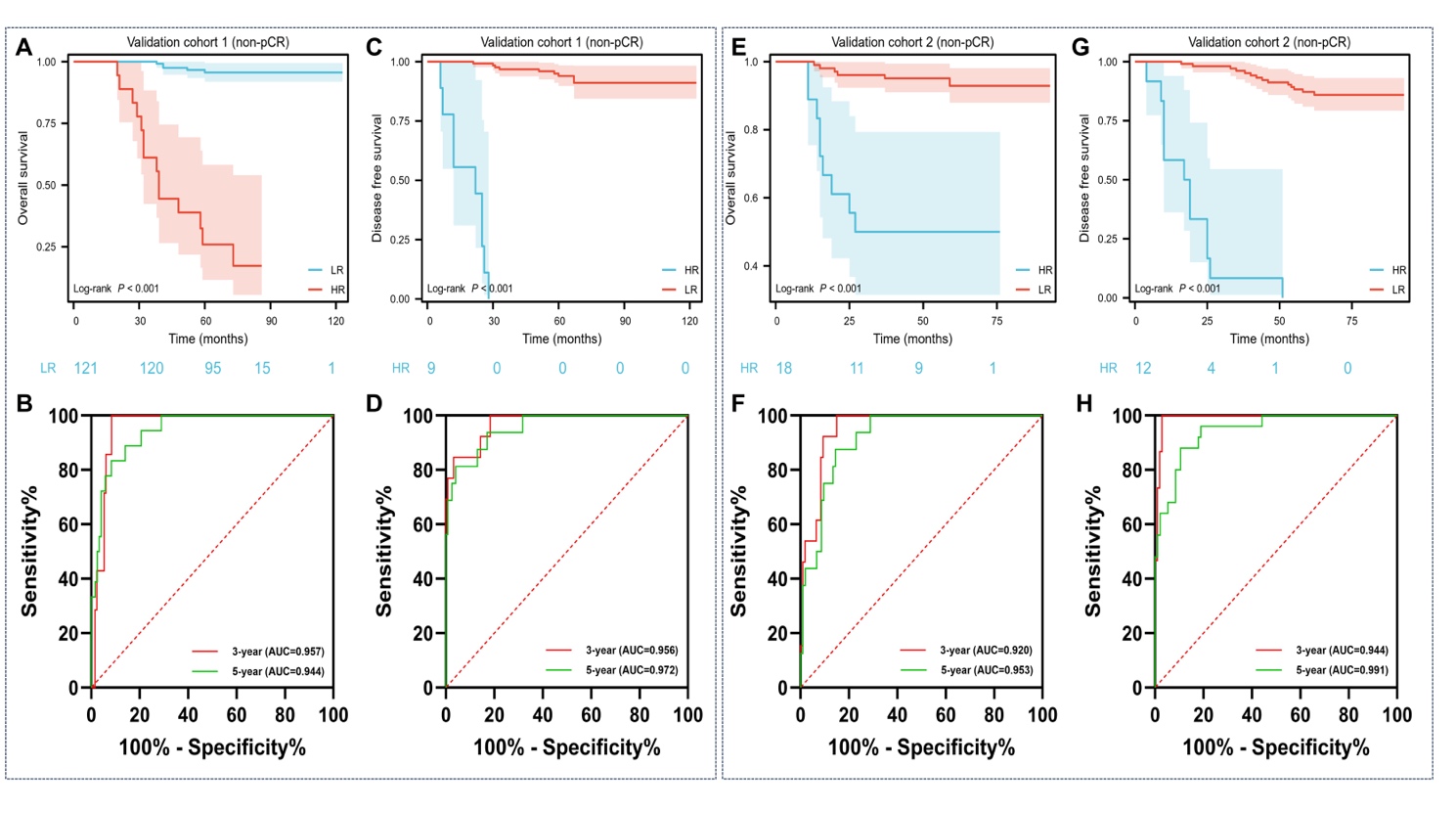


**FIGURE S3.** (A, E) Kaplan–Meier curves for OS stratified by CIOPM risk groups in non-pCR subtypes in VC 1 and VC 2; (C, G) Kaplan–Meier curves for DFS stratified by CIOPM risk groups in in non-pCR subtypes in VC 1 and VC 2; (B, F) Time-dependent ROC curves for 3-year and 5-year OS prediction using CIOPM in non-pCR subtypes in VC 1 and VC 2; (D, H) Time-dependent ROC curves for 3-year and 5-year DFS prediction using CIOPM in non-pCR subtypes in VC 1 and VC 2. Abbreviations: CIOPM, ClinicHistomics Integrated Outcome Prediction Model; OS, overall survival; DFS, disease-free survival; VC, Validation Cohort; ROC, receiver operating characteristic.


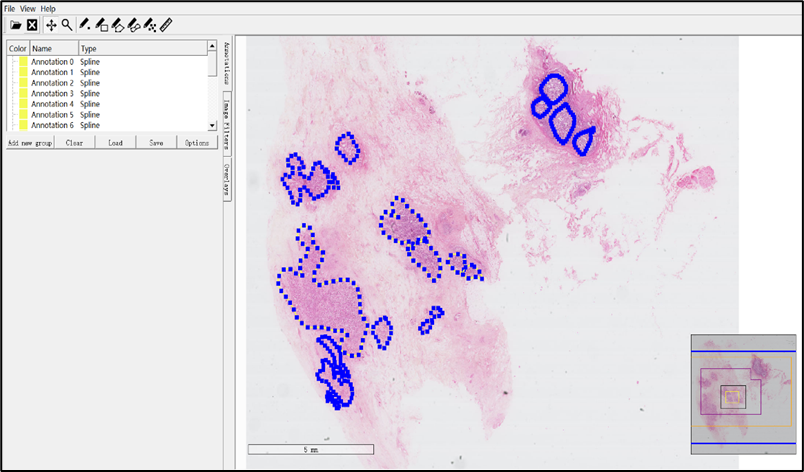


**FIGURE S4** The working interface of ASAP 1.9.0 software and the tumor area labelling. The blue line in the figure shows the outlined tumor area, and the number of labelled traces is shown in the upper left corner.


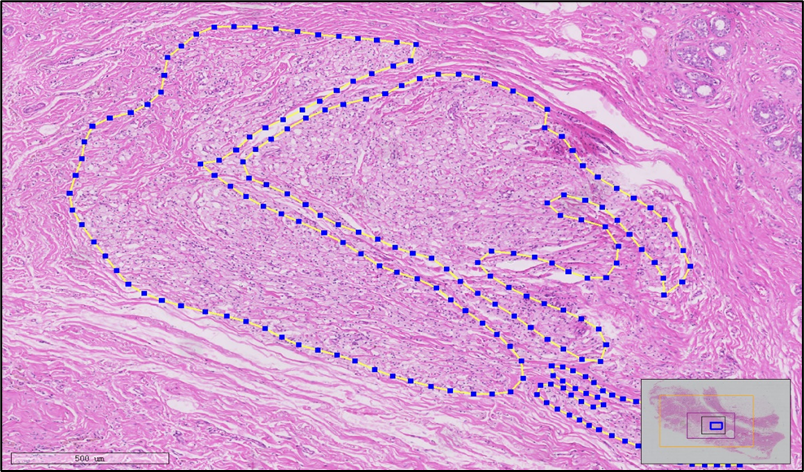


**FIGURE S5 The labelling process for tumor beds.** The blue dots and yellow lines in the figure show the location of the tumor bed (foam cells).


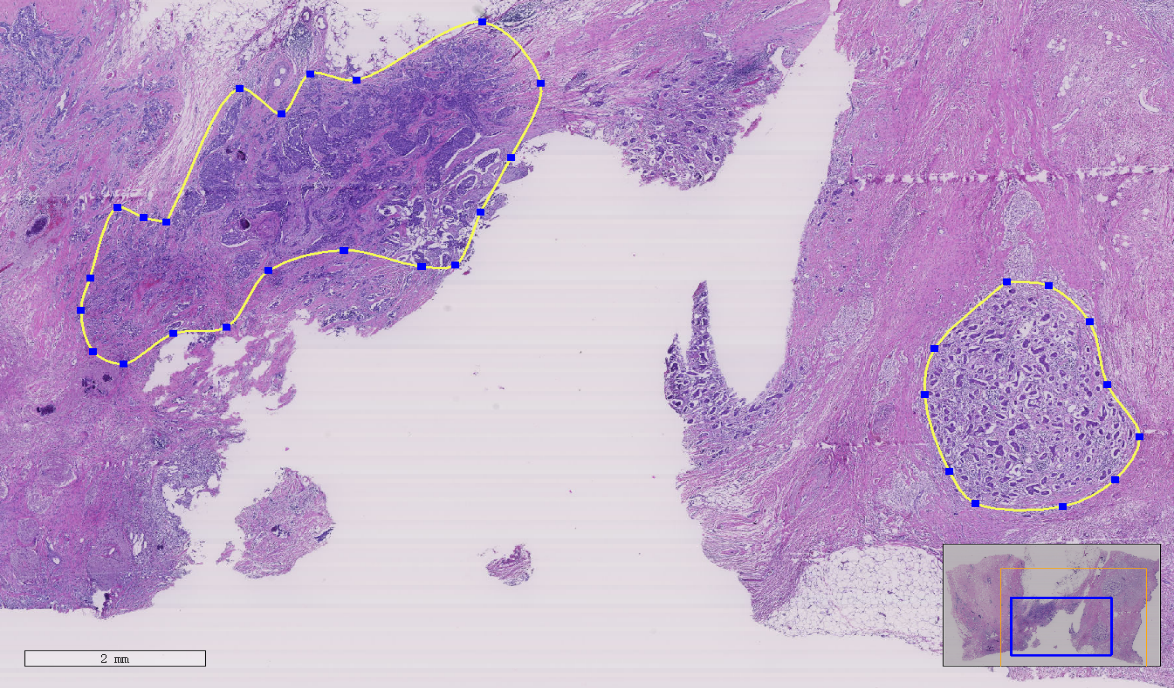


**FIGURE S6 The labelling process for heterogeneous tumors.** The marked areas of heterogeneous tumors are indicated by the blue dots and yellow lines in the figure. It is clear from the figure that the histological patterns of the region of interests on the left and right differ significantly.

| **TABLE S1** The results of the ablation study. | | | | |
| --- | --- | --- | --- | --- |
| **Models** | | **C-index (95% CI)** | | |
|  |  | **CIM** | **PIM** | **CIOPM** |
| **VC 1**  **(n=142)** | **OS** | 0.873 (0.782-0.921) | 0.687 (0.583-0.768) | 0.933 (0.878-0.977) |
|  | **DFS** | 0.901 (0.868-0.938) | 0.597 (0.475-0.644) | 0.947 (0.895-0.983) |
| **VC 2**  **(n=143)** | **OS** | 0.853 (0.819-0.897) | 0.637 (0.498-0.748) | 0.915 (0.850-0.960) |
|  | **DFS** | 0.849 (0.804-0.885) | 0.646 (0.537-0.752) | 0.937 (0.905-0.965) |
| Abbreviations: VC, validation cohort; CI, Confidence Interval; OS, overall survival; DFS, disease free survival; CIM, the Clinical Information Model; PIM, pathologic Image Model; CIOPM, the ClinicHistomics Integrated Outcome Prediction Model. | | | | |

| **TABLE S4** Summary of univariate and multivariate Cox model estimates for OS (CIOPM) on VC 1. | | |
| --- | --- | --- |
| **Characteristics** | **Univariate Cox analysis** | **Multivariate Cox analysis** |
|  | ***P* (HR, 95% CI)** | ***P* (HR, 95% CI)** |
| **Age (≤50 vs. ＞50)** | 0.647 (1.32, 0.40-4.33) | - |
| **Menstrual status (N vs. Y)** | 0.165 (0.43, 0.13-1.41) | - |
| **Laterality (L vs. R)** | 0.652 (1.32, 0.39-4.56) | - |
| **CIOPM_OS_ score** | **<0.001** (480.00, 42.32-5.45×10^3^) | **0.001 (**1.18×10^4^, 53.88-2.62×10^5^) |
| **NAT type** | 0.703 | - |
| anthracycline | Ref | - |
| paclitaxel | 0.994 (1.07, 0.00-1.04×10^7^) | - |
| anthracycline and paclitaxel | 0.490 (26.68, 0.00-3.01×10^5^) | - |
| **non-pCR vs. pCR** | 0.125 (0.20, 0.03-1.57) | 0.161 (0.04, 0.00-3.81) |
| **cT (T3, T4 vs. T1, T2)** | 0.081 (3.00, 0.87-10.33) | 0.912 (0.87, 0.08-9.66) |
| **cN (N2, N3 vs. N0, N1)** | 0.303 (1.92, 0.56-6.59) | - |
| **pT** | **0.043** | 0.060 |
| pT0 | Ref | - |
| pT1+pT2 | 0.012 (0.14, 0.03-0.65) | 0.018 (0.01, 0.00-0.46) |
| pT3+pT4 | 0.988 (0.00, -) | 0.987 (0.00, -) |
| **pN** | 0.810 | 0.212 |
| pN0 | Ref | Ref |
| pN1+pN2 | 0.620 (1.50, 0.30-7.50) | 0.214 (0.11, 0.00-0.46) |
| pN3 | 0.520 (1.80, 0.30-10.91) | 0.728, (0.00, -) |
| **ER (pre)** (NE vs. PO) | **0.042** (0.29, 0.09-0.96) | - |
| **PR (pre)** (NE vs. PO) | **0.044** (0.29, 0.09-0.97) | - |
| **HER2 (pre)** (NE vs. PO) | 0.195 (0.36, 0.08-1.69) | 0.081 (7.48, 0.78-71.47) |
| **Ki67 (pre) (﹥20% vs. ≦20%)** | 0.606 (1.72, 0.22-13.43) | 0.547 (2.21, 0.17-29.15) |
| **Subtype (pre)** | **0.006** | - |
| HR+/HER2- | Ref | - |
| HR+/HER2+ | 0.003 (72.88, 4.13-1.29×10^3^) | - |
| HR-/HER2+ | 0.420 (2.42, 0.28-20.81) | - |
| HR-/HER2- | 0.009 (5.76, 1.54-21.62) | - |
| **LVI (post) (present vs. absent)** | 0.264 (2.02, 0.59-6.91) | 0.619 (1.76, 0.19-16.57) |
| **Radiotherapy (Y vs. N)** | 0.310 (0.54, 0.17-1.79) | - |
| **Endocrine therapy (Y vs. N)** | **0.035** (0.24, 0.06-0.90) | - |
| **Surgery type (M vs. L)** | 0.711 (0.05, 0.00-4.62×10^5^) | - |
| OS, overall survival; HR, hazard ratio; Y, yes; N, no; L, left; R, right; NAT, neoadjuvant chemotherapy; pCR, pathology complete response; cT, clinical tumour stage; cN, clinical N stage; pT, pathologic T stage; pN, pathologic N stage; NE, negative; PO, positive; TNBC, triple-negative breast cancer; LVI, lymphvascular invasion; M, Mastectomy; L, Lumpectomy. | | |

| **TABLE S5** Summary of univariate and multivariate Cox model estimates for DFS (CIOPM) on VC 1. | | |
| --- | --- | --- |
| **Characteristics** | **Univariate Cox analysis** | **Multivariate Cox analysis** |
|  | ***P* (HR, 95% CI)** | ***P* (HR, 95% CI)** |
| **Age (≤50 vs. ＞50)** | 0.503 (1.17, 0.75-1.82) | - |
| **Menstrual status (N vs. Y)** | 0.611 (0.89, 0.58-1.37) | - |
| **Laterality (L vs. R)** | 0.879 (0.97, 0.64-1.47) | - |
| **CIOPM_DFS_ score** | **<0.001** (1.27, 1.23-1.32) | **<0.001** **(**1.27, 1.22-1.33) |
| **NAT type** | 0.979 | - |
| anthracycline | Ref | - |
| paclitaxel | 0.839 (0.91, 0.43-2.35) | - |
| anthracycline and paclitaxel | 0.857 (0.93, 0.43-2.03) | - |
| **non-pCR vs. pCR** | 0.198 (2.51, 0.62-10.21) | - |
| **cT (T3, T4 vs. T1, T2)** | **0.048** (1.56, 1.00-2.42) | 0.609 (1.14, 0.69-1.87) |
| **cN (N2, N3 vs. N0, N1)** | **0.001** (2.19, 1.38-3.47) | 0.879 (1.04, 0.62-1.76) |
| **pT** | **<0.001** | 0.878 |
| pT0 | Ref | Ref |
| pT1+pT2 | 0.192 (2.55, 0.63-10.44) | 0.953 (1.05, 0.24-4.63) |
| pT3+pT4 | 0.004 (8.17, 1.92-34.79) | 0.611 (1.50, 0.31-7.18) |
| **pN** | **<0.001** | 0.878 |
| pN0 | Ref | Ref |
| pN1+pN2 | 0.278 (1.36, 0.78-2.39) | 0.610 (0.85, 0.45-1.60) |
| pN3 | <0.001 (3.03, 1.64-5.58) | 0.706, (0.87, 0.42-1.80) |
| **ER (pre)** (NE vs. PO) | 0.925 (1.02, 0.63-1.67) | - |
| **PR (pre)** (NE vs. PO) | 0.380 (1.22, 0.78-1.92) | - |
| **HER2 (pre)** (NE vs. PO) | **0.041** (0.61, 0.38-0.98) | 0.986 (0.99, 0.30-3.32) |
| **Ki67 (pre) (﹥20% vs. ≦20%)** | 0.566 (1.18, 0.67-2.11) | 0.547 (2.21, 0.17-29.15) |
| **Subtype (pre)** | 0.094 | 0.941 |
| HR+/HER2- | Ref | Ref |
| HR+/HER2+ | 0.013 (1.92, 1.15-3.21) | 0.836 (1.14, 0.33-4.02) |
| HR-/HER2+ | 0.985 (0.99, 0.31-3.17) | - |
| HR-/HER2- | 0.393 (1.31, 0.71-2.40) | 0.778 (0.91, 0.46-1.78) |
| **LVI (post) (present vs. absent)** | **<0.001** (2.32, 1.49-3.63) | 0.626 (0.88, 0.52-1.49) |
| **Radiotherapy (Y vs. N)** | 0.222 (1.33, 0.84-2.10) | - |
| **Endocrine therapy (Y vs. N)** | 0.760 (0.93, 0.57-1.51) | - |
| **Surgery type (M vs. L)** | 0.639 (1.40, 0.34-5.69) | - |
| DFS, disease free survival; HR, hazard ratio; Y, yes; N, no; L, left; R, right; NAT, neoadjuvant chemotherapy; pCR, pathology complete response; cT, clinical tumour stage; cN, clinical N stage; pT, pathologic T stage; pN, pathologic N stage; NE, negative; PO, positive; TNBC, triple-negative breast cancer; LVI, lymphvascular invasion; M, Mastectomy; L, Lumpectomy. | | |

| **TABLE S6** Summary of univariate and multivariate Cox model estimates for OS (CIOPM) on VC2. | | |
| --- | --- | --- |
| **Characteristics** | **Univariate Cox analysis** | **Multivariate Cox analysis** |
|  | ***P* (HR, 95% CI)** | ***P* (HR, 95% CI)** |
| **Age** **(≤50 vs. ＞50)** | 0.827 (1.05, 0.39-2.79) | - |
| **Menstrual status (N vs. Y)** | 0.977 (0.99, 0.35-2.76) | - |
| **Laterality (L vs. R)** | 0.546 (0.75, 0.30-1.90) | - |
| **CIOPM_OS_ score** | **<0.001** (186.53, 38.61-901.15) | **<0.001 (**147.96, 12.04-1.82×10^3^) |
| **NAT type** | 0.126 | - |
| anthracycline | Ref | - |
| paclitaxel | 0.433 (0.46, 0.64-3.24) | - |
| anthracycline and paclitaxel | 0.054 (0.23, 0.05-1.03) | - |
| **non-pCR vs. pCR** | 0.549 (1.57, 0.36-6.82) | - |
| **cT (T3, T4 vs. T1, T2)** | 0.026 (2.87, 1.13-7.28) | - |
| **cN (N2, N3 vs. N0, N1)** | 0.998 (1.00, 0.38-2.58) | - |
| **pT** | **<0.001** | **0.016** |
| pT0 | Ref | Ref |
| pT1+pT2 | 0.998 (1.00, 0.22-4.62) | 0.355 (0.45, 0.08-2.45) |
| pT3+pT4 | 0.003 (11.24, 2.31-54.62) | 0.415 (2.19, 0.33-14.50) |
| **pN** |  | - |
| pN0 | Ref | - |
| pN1+pN2 | 0.223 (1.90, 0.68-5.33) | - |
| pN3 | - | - |
| **ER (pre)** (NE vs. PO) | 0.081 (2.29, 0.90-5.80) | - |
| **PR (pre)** (NE vs. PO) | **0.014** (3.63, 1.29-10.19) | 0.508 (0.61, 0.14-2.68) |
| **HER2 (pre)** (NE vs. PO) | 0.317 (1.69, 0.60-4.75) | - |
| **Ki67 (pre) (﹥20% vs. ≦20%)** | 0.646 (1.34, 0.39-4.62) | - |
| **Subtype (pre)** | **0.004** | - |
| HR+/HER2- | Ref | - |
| HR+/HER2+ | 0.116 (2.87, 0.77-10.69) | - |
| HR-/HER2+ | 0.969 (-, 0.00-2.43E+250) | - |
| HR-/HER2- | <0.001 (8.58, 2.64-27.93) | - |
| **LVI (post) (present vs. absent)** | 0.388 (1.63, 0.54-4.96) | - |
| **Radiotherapy (Y vs. N)** | 0.904 (1.07, 0.38-2.99) | - |
| **Endocrine therapy (Y vs. N)** | **0.002** (0.14, 0.04-0.50) | 0.451 (0.54, 0.11-2.69) |
| **Surgery type (M vs. L)** | - | - |
| OS, overall survival; HR, hazard ratio; Y, yes; N, no; L, left; R, right; NAT, neoadjuvant chemotherapy; pCR, pathology complete response; cT, clinical tumour stage; cN, clinical N stage; pT, pathologic T stage; pN, pathologic N stage; NE, negative; PO, positive; TNBC, triple-negative breast cancer; LVI, lymphvascular invasion; M, Mastectomy; L, Lumpectomy. | | |

| **TABLE S7** Summary of univariate and multivariate Cox model estimates for DFS (CIOPM) on VC2. | | |
| --- | --- | --- |
| **Characteristics** | **Univariate Cox analysis** | **Multivariate Cox analysis** |
|  | ***P* (HR, 95% CI)** | ***P* (HR, 95% CI)** |
| **Age (≤50 vs. ＞50)** | 0.890 (1.06, 0.49-2.26) | - |
| **Menstrual status (N vs. Y)** | 0.980 (1.01, 0.45-2.27) | - |
| **Laterality (L vs. R)** | 0.619 (0.83, 0.41-1.71) | - |
| **CIOPM_DFS_ score** | **<0.001** (3.11×10^3^, 477.90-2.02× 10⁴) | **<0.001** (1.58×10⁴, 1.28×10³-1.96×10⁵) |
| **NAT type** | 0.703 | - |
| anthracycline | Ref | - |
| paclitaxel | 0.755 (1.43, 0.15-13.80) | - |
| anthracycline and paclitaxel | 0.884 (0.86, 0.12-6.36) | - |
| **non-pCR vs. pCR** | 0.612 (1.31, 0.46-3.76) | - |
| **cT (T3, T4 vs. T1, T2)** | 0.069 (1.95, 0.95-4.03) | - |
| **cN (N2, N3 vs. N0, N1)** | 0.872 (1.06, 0.51-2.20) | - |
| **pT** | **<0.001** | 0.063 |
| pT0 | Ref | Ref |
| pT1+pT2 | 0.898 (1.07, 0.37-3.15) | 0.066 (0.32, 0.09-1.08) |
| pT3+pT4 | 0.010 (7.59, 2.20-26.19) | 0.023 (11.10, 0.11-0.71) |
| **pN** | - | - |
| pN0 | Ref | - |
| pN1+pN2 | 0.013 (3.10, 1.27-7.59) | - |
| pN3 | - | - |
| **ER (pre)** (NE vs. PO) | 0.782 (1.11, 0.53-2.34) | - |
| **PR (pre)** (NE vs. PO) | 0.259 (1.51, 0.74-3.09) | - |
| **HER2 (pre)** (NE vs. PO) | 0.066 (2.21, 0.95-5.15) | - |
| **Ki67 (pre) (﹥20% vs. ≦20%)** | 0.417 (0.72, 0.32-1.61) | - |
| **Subtype (pre)** | 0.131 | - |
| HR+/HER2- | Ref | - |
| HR+/HER2+ | 0.317 (0.57, 0.19-1.71) | - |
| HR-/HER2+ | 0.240 (0.48, 0.14-1.64) | - |
| HR-/HER2- | 0.167 (1.87, 0.77-4.56) | - |
| **LVI (post) (present vs. absent)** | **0.009** (2.82, 1.29-6.17) | 0.540 (1.33, 0.53-3.31) |
| **Radiotherapy (Y vs. N)** | 0.245 (1.70, 0.70-4.16) | - |
| **Endocrine therapy (Y vs. N)** | 0.055 (0.49, 0.24-1.02) | - |
| **Surgery type (M vs. L)** | - | - |
| OS, overall survival; HR, hazard ratio; Y, yes; N, no; L, left; R, right; NAT, neoadjuvant chemotherapy; pCR, pathology complete response; cT, clinical tumour stage; cN, clinical N stage; pT, pathologic T stage; pN, pathologic N stage; NE, negative; PO, positive; TNBC, triple-negative breast cancer; LVI, lymphvascular invasion; M, Mastectomy; L, Lumpectomy. | | |

| **TABLE S8** The details of the antibodies | | | | | |
| --- | --- | --- | --- | --- | --- |
| **Antibody name** | **Company** | **Clone number** | **Repair conditions** | **Dilution ratio** | **Species** |
| ER | Abcam | ab32063 | pH 9 (EDTA) | 1:200 | rabbit |
| PR | Abcam | ab32085 | pH 9 (EDTA) | 1:100 | rabbit |
| HER2 | Roche | 4B5 | pH 9 (EDTA) | no dilution | rabbit |
| Ki67 | Roche | 30-9 | pH 9 (EDTA) | no dilution | rabbit |
| ER, estrogen receptor; PR, progesterone receptor; HER2, human epidermal growth factor receptor-2. | | | | | |
|  |  |  |  |  |  |

| **TABLE S9** Point Assignments for the yAJCC, RCB and Neo-Bioscore Staging Systems | | | |
| --- | --- | --- | --- |
| Staging Systems | Abbreviation | Categories | Factors included |
| American Joint Committee Cancer | yAJCC | 0, Ⅰ (a-b), Ⅱ (a-b), Ⅲ (a-c), Ⅳ | T-tumor size, invasion of local structures  N-nodal metastases  M-distant metastases |
| Residual cancer burden | RCB | 0, Ⅰ, Ⅱ, Ⅲ | Primary tumor bed dimensions (√d1d2)  Cellularity fraction of invasive cancer (finv)  Size of largest metastasis (dmet)  Number of positive lymph nodes |
| Neo-Bioscore | - | 0~7 | Clinical stage, pathologic stage, ER negative, Grade 3, ERBB2 negative |
| RCB, Residual Cancer Burden; AJCC, The eighth edition American Joint Committee on Cancer; NAT, neoadjuvant therapy; finv, cellularity fraction of invasive cancer; pT, pathologic tumor size; pN, pathologic lymph node; M, metastasis; cTNM, clinical stage. | | | |
